# Hepatocyte-specific Prominin-1 protects against liver injury-induced fibrosis by stabilizing SMAD7

**DOI:** 10.1038/s12276-022-00831-y

**Published:** 2022-08-29

**Authors:** Hyun Lee, Dong-Min Yu, Myeong-Suk Bahn, Young-Jae Kwon, Min Jee Um, Seo Yeon Yoon, Ki-Tae Kim, Myoung-Woo Lee, Sung-Je Jo, Sungsoo Lee, Seung-Hoi Koo, Ki Hoon Jung, Jae-Seon Lee, Young-Gyu Ko

**Affiliations:** 1 Tunneling Nanotube Research Center, Korea University, Seoul, 02841 Korea; 2 Division of Life Sciences, Korea University, Seoul, 02841 Korea; 3 Department of Surgery, Dongguk University College of Medicine, Gyeongju, 38067 Korea; 4 Research Center for Controlling Intercellular Communication, College of Medicine, Inha University, Incheon, 22212 Korea

**Keywords:** Cell signalling, Apoptosis, Post-translational modifications, Mechanisms of disease

## Abstract

Prominin-1 (PROM1), also known as CD133, is expressed in hepatic progenitor cells (HPCs) and cholangiocytes of the fibrotic liver. In this study, we show that PROM1 is upregulated in the plasma membrane of fibrotic hepatocytes. Hepatocellular expression of PROM1 was also demonstrated in mice (*Prom1*^*CreER*^*; R26*^*TdTom*^) in which cells expressed TdTom under control of the *Prom1* promoter. To understand the role of hepatocellular PROM1 in liver fibrosis, global and liver-specific *Prom1*-deficient mice were analyzed after bile duct ligation (BDL). BDL-induced liver fibrosis was aggravated with increased phosphorylation of SMAD2/3 and decreased levels of SMAD7 by global or liver-specific *Prom1* deficiency but not by cholangiocyte-specific *Prom1* deficiency. Indeed, PROM1 prevented SMURF2-induced SMAD7 ubiquitination and degradation by interfering with the molecular association of SMAD7 with SMURF2. We also demonstrated that hepatocyte-specific overexpression of SMAD7 ameliorated BDL-induced liver fibrosis in liver-specific *Prom1-*deficient mice. Thus, we conclude that PROM1 is necessary for the negative regulation of TGFβ signaling during liver fibrosis.

## Liver disease: Preventing progression of fibrosis

Progression of liver fibrosis is kept in check by a regulatory protein that switches off a signaling pathway responsible for cell death and subsequent scar tissue formation. Liver fibrosis is a common outcome of alcoholism, viral infection, and hepatitis. Researchers led by Young-Gyu Ko at Korea University, Seoul, South Korea, determined that a protein called PROM-1 is highly expressed in fibrotic liver tissue from mice and humans, and set out to uncover its function. They found that PROM-1 exerts a protective role, as PROM-1-deficient mice experienced accelerated liver degeneration in response to bile duct injury. PROM-1 acts by blocking the effects of transforming growth factor-β, a signaling protein which promotes cell death. These results are consistent with evidence linking PROM-1 to anti-fibrotic activity in other organ systems.

## Introduction

Liver fibrosis results from the accumulation of collagenous scar tissue following chronic liver injury caused by nonalcoholic steatohepatitis (NASH), alcohol, hepatitis viral infection, and autoimmune hepatitis^1,2^. Chronic liver fibrosis progresses into hepatocellular carcinoma and cirrhosis, characterized by ascites, jaundice (yellow skin), hepatic hypertension, and hepatic encephalopathy, if the cause is not eliminated^3^. Liver fibrosis is initiated by damaged hepatocytes and infiltrated macrophages, which secrete profibrogenic cytokines such as transforming growth factor-β (TGFβ), platelet-derived growth factor (PDGF), connective tissue growth factor (CTGF), vascular endothelial growth factor (VEGF), sonic hedgehog (SHH) and WNT4. Hepatic stellate cells (HSCs) residing between hepatocytes and endothelial cells proliferate and are activated by these profibrogenic cytokines to produce extracellular matrix (ECM) components, such as collagen and fibronectin^4–6^.

TGFβ binds its type II receptor (TβRII), recruiting and phosphorylating its type I receptor (TβRI) in HSCs. Activated TβRI induces the phosphorylation of SMAD2 and SMAD3 and activated SMAD2/3 then bind SMAD4. The SMAD2/3-SMAD4 complex enters the nucleus and acts as a transcription factor to upregulate the genes encoding type I collagens (*COL1A1* and *COL1A2*), tissue inhibitors of matrix metalloproteases (*TIMPs*), and α-smooth muscle actin (*ACTA2*)^7,8^. The activated SMAD2/3-SMAD4 complex also upregulates SMAD7, a negative feedback inhibitor of TGFβ signaling. SMAD7 binds TβRI and induces the ubiquitination and degradation of TβRI by recruiting SMAD ubiquitination regulatory factors 1 and 2 (SMURF1 and 2), thereby preventing TGFβ-induced phosphorylation and activation of SMAD2/3^9–11^. SMAD7 also prevents the phosphorylation of SMAD3 or formation of the SMAD2/3-SMAD4 complex without TβRI ubiquitination^12,13^.

TGFβ plays important role in hepatocytes during liver fibrosis^7,8^. For example, hepatocyte-specific *Tgfbr2* deletion or *Smad7* overexpression prevents liver fibrosis development via NASH, bile duct ligation (BDL), and carbon tetrachloride (CCl_4_)^14–16^. The transdifferentiation of primary cultured hepatocytes into myofibrils is also prevented by adenoviral overexpression of *Smad7*^14^.

Prominin-1 (PROM1), also called CD133, is a pentaspan transmembrane glycoprotein expressed in hematopoietic, epithelial, and intestinal progenitor cells^17,18^. PROM1 is expressed in rat HSCs and has been used as a cell-surface marker to isolate hepatic progenitor cells (HPCs)^19^. High expression levels of PROM1 are also observed in HPCs and cholangiocytes after liver injury, such as that due to rhesus rotavirus (RRV)-induced biliary atresia (BA) and BDL^20,21^. Because *Prom1* deficiency was shown to protect against RRV-induced liver fibrosis in mice, the upregulation of *Prom1* in HPCs might be necessary to promote biliary fibrosis^22^.

In this study, we demonstrate the expression of PROM1 in the hepatocyte plasma membrane as well as in HPCs and cholangiocytes. We also demonstrate that PROM1 deficiency aggravated BDL-induced liver fibrosis and enhanced TGFβ signaling by reducing SMAD7 protein expression in hepatocytes. Because PROM1 interfered with the molecular association of SMAD7 with SMURF2 and prevented SMURF2-induced SMAD7 ubiquitination and degradation, we conclude that PROM1 is necessary for the prevention of liver fibrosis by negatively regulating TGFβ signaling.

## Materials and methods

### Patient samples

Samples from human patients with mild or severe fibrosis were obtained from Dongguk University in Gyeongju, South Korea (approval number: 110757-201909-HR-06-02). The patients had been diagnosed with liver fibrosis or cirrhosis by histological examination at hospitals in South Korea.

### Animal models and experiments

Global *Prom1* knockout (KO) mice (The Jackson Laboratory, Bar Harbor, ME, USA), in which the ATG start codon of the CD133 gene was replaced with a CreERT2 fusion protein and the IRES-β-galactosidase (lacZ) gene, were utilized. *Prom1* KO mice were backcrossed onto a C57BL/6N background for at least five generations. To generate liver-specific *Prom1*-deficient mice (*Prom1*^*f/f*^*; Alb-Cre*), *Prom1*^*f/f*^ mice (*f/f*) were created by ToolGen (Seoul, Korea) and crossed with Alb-Cre (*Alb-Cre*) mice containing a *Cre* recombinase driven by an albumin promoter (The Jackson Laboratory, Bar Harbor, ME, USA).

We generated *f/f; Krt19-Cre* mice by crossing *Krt19-Cre* mice (stock No: 026925, The Jackson Laboratory) with *Prom1*^*f/f*^ mice. For Cre-loxP recombination, tamoxifen (T5648; Sigma, USA) at a concentration of 20 mg/ml in corn oil was injected intraperitoneally at 100 mg/kg body weight for 5 consecutive days into 6- to 8-week-old mice. To generate *Krt19*^*CreER*^*; Prom1*^*f/f*^*; R26*^*TdTom*^ mice, *Krt19*^*CreER*^ (stock No: 026925, The Jackson Laboratory), *R26*^*TdTom*^ (stock No: 007914, The Jackson Laboratory), and *Prom1*^*f/f*^ mice were mated with each other. For Cre-loxP recombination, tamoxifen (T5648; Sigma) at a concentration of 20 mg/ml in corn oil was injected intraperitoneally at 100 mg/kg bodyweight for 5 consecutive days into 6- to 8-week-old mice.

*Prom1* cell lineage tracing mice (*Prom1*^*CreER*^*; R26*^*TdTom*^) were generated by crossing *Prom1*^*CreER*^ mice (stock No: 017743, The Jackson Laboratory) and *R26*^*TdTom*^ mice (stock No: 007914, The Jackson Laboratory). To achieve Cre-loxP recombination, tamoxifen (T5648; Sigma) at a concentration of 20 mg/ml in corn oil was injected once intraperitoneally at 150 mg/kg body weight one day after BDL into 8- to 10-week-old mice.

Mice, housed in plastic cages under a 12 h:12 h light-dark photoperiod with free access to water and food, were bred, maintained, and cared for in a manner consistent with criteria outlined in the Principles of Laboratory Animal Care (NIH publication no. 85-23, revised 1985), and protocols were approved by the Institutional Animal Care and Use Committee of Korea University. Notably, *Prom1* KO mice are viable and live to adulthood without exhibiting phenotypic differences from their wild-type littermates. All animal studies were conducted with the approval of the Korea University Institutional Animal Care and Use Committee and the Korean Animal Protection Law (KUIACUC-2018-06 and KUIACUC-2019-0111).

### Mouse model of liver fibrosis

To generate different mouse fibrosis models, we subjected mice to BDL. For the BDL model, the mice were randomly divided into two groups: animals that underwent BDL and sham-operated animals that were used as healthy controls. Briefly, mice were anesthetized using isoflurane. The extrahepatic bile duct was isolated and doubly ligated. The peritoneal cavity was filled with saline, and the incisions were closed. Mouse body weight was measured daily. Seven days after surgery, BDL and sham control animals were sacrificed.

### Adenovirus preparation and infection

Adenoviruses harboring LacZ, PROM1, GFP, and SMAD7 were produced as previously described^23^. Each viral stock was infected into AD293 cells to amplify the viruses. Viruses amplified in AD293 cells were harvested and purified by double cesium chloride‐gradient ultracentrifugation. Infectious viral particles were collected using a syringe, washed with washing buffer (10 mM Tris (pH 8.0), 2 mM MgCl_2_, and 5% sucrose), and stored at −80 °C. Recombinant adenovirus (0.5 × 10^9^ pfu) was injected into the tail veins of mice.

### Serum biochemistry

Serum alanine aminotransferase (ALT), aspartate aminotransferase (AST), and total bilirubin levels were analyzed using a spectraMAX (Molecular Devices) and commercial kits from BioVision (San Francisco, USA). Serum levels were analyzed using commercial kits according to the manufacturer’s instructions.

### Immunohistochemistry

Freshly frozen tissues postfixed in 4% paraformaldehyde were subjected to immunohistochemistry. Tissue sections were immunostained with antibodies against PROM1 (Developmental Studies Hybridoma Bank, eBioscience or Miltenyi Biotec), CK19, αSMA, SMAD7, p-SMAD2/3, and SMAD2/3. Briefly, the sections were pretreated with 2.5% horse serum for 20 min to block nonspecific antibody binding and then incubated with the antibodies of interest overnight at 4 °C. Supplementary Table 1 shows the antibodies used for immunostaining. The slides were then treated with a FITC- or rhodamine-conjugated secondary antibody. After mounting with Permount solution, the samples were imaged on an LSM 800 META confocal microscope (Carl Zeiss, Thornwood, NY).

### Western blotting

Immunoblot analysis was performed as previously described. Briefly, cells were harvested with lysis buffer (25 mM HEPES, 150 mM NaCl, 1% NP-40, 10 mM MgCl_2_, and 1 mM EDTA, pH 7.5) on ice for 20 min. The lysates were centrifuged at 10,000 × *g* for 10 min to obtain supernatants. Proteins were separated by SDS–polyacrylamide gel electrophoresis, and the immobilized proteins were immunoblotted with the antibodies of interest. Supplementary Table 1 shows the antibodies used for immunoblotting analysis. The antigens were visualized using an ECL substrate kit (Thermo Scientific, USA).

For immunoprecipitation, cells were lysed in buffer containing 20 mM Tris-HCl (pH 7.4), 137 mM NaCl, 1 mM MgCl_2_, 1 mM CaCl_2_, and a protease inhibitor cocktail (Sigma-Aldrich, St Louis, MO, USA). Whole-cell lysates (500 µg of protein) were incubated with specific antibodies overnight and then with 60 μl of a slurry of Protein A-agarose or Protein G-agarose beads (Roche, Mannheim, Germany) for 3 h. Immunoprecipitants were analyzed by immunoblotting.

### Preparation of mouse primary hepatocytes

Primary hepatocytes (PHs) were isolated from 8-week-old C57BL/6 male mice as previously described. Briefly, mice were anesthetized with avertin (intraperitoneal injection of 250 mg/kg body weight), and livers were perfused with a preperfusion buffer (140 mM NaCl, 6 mM KCl, 10 mM HEPES, and 0.08 mg/mL EGTA, pH 7.4) at a rate of 7 mL/min for 5 min, followed by continuous perfusion with a collagenase-containing buffer (66.7 mM NaCl, 6.7 mM KCl, 5 mM HEPES, 0.48 mM CaCl_2_, and 3 g/mL collagenase type IV, pH 7.4) for 8 min. Viable hepatocytes were harvested and purified with a Percoll gradient. Then, hepatocytes were resuspended in complete growth medium (199 medium containing 10% FBS, 23 mM HEPES and 10 nM dexamethasone) and seeded on collagen-coated plates at a density of 300,000 cells/ml. After a 4-h attachment period, the medium was replaced with complete growth medium before the hepatocytes were used in any experiments and was changed daily.

### Quantitative real-time PCR

RNA (2 μg) was reverse transcribed into cDNA using random hexamer primers, oligo(dT), and reverse transcription master premix (ELPIS Biotech, Daejeon, Korea). Quantitative real-time PCR was performed using cDNA from the reverse transcription reactions and gene-specific oligonucleotides in the presence of TOPreal qPCR 2X premix (Enzynomics, Daejeon, Korea). The following PCR conditions were used: an initial denaturation step at 95 °C for 10 min, followed by 45 cycles of denaturation at 95 °C for 10 s, annealing at 58 °C for 15 s, and elongation at 72 °C for 20 s. The melting curve for each PCR product was assessed for quality control. Supplementary Table 2 shows the sequences of the primers used for qPCR.

### TUNEL assay

To detect apoptotic cells in the livers, we performed terminal deoxynucleotidyl transferase dUTP nick-end labeling (TUNEL) according to the manufacturer’s protocol (Promega, Madison, Wisconsin, USA). Briefly, paraffin-embedded liver tissues were prepared on slides. Fluorescein-12-dUTP (a) was catalytically incorporated into the fragmented DNA of apoptotic cells at 3′-OH DNA ends using recombinant terminal deoxynucleotidyl transferase (rTdT). Counterstaining of the liver nuclei was carried out with DAPI for 10 min. The fluorescein-12-dUTP-labeled DNA was then either visualized directly by confocal microscopy (LSM 800 Carl Zeiss, Germany).

### Statistical analysis

Statistical values are presented as the mean ± S.E.M. A two-tailed *t* test was used to compare groups (*p < 0.05, **p < 0.01, ***p < 0.001).

## Results

### PROM1 is upregulated in hepatocytes and cholangiocytes of the fibrotic liver

**Fig. 1:**
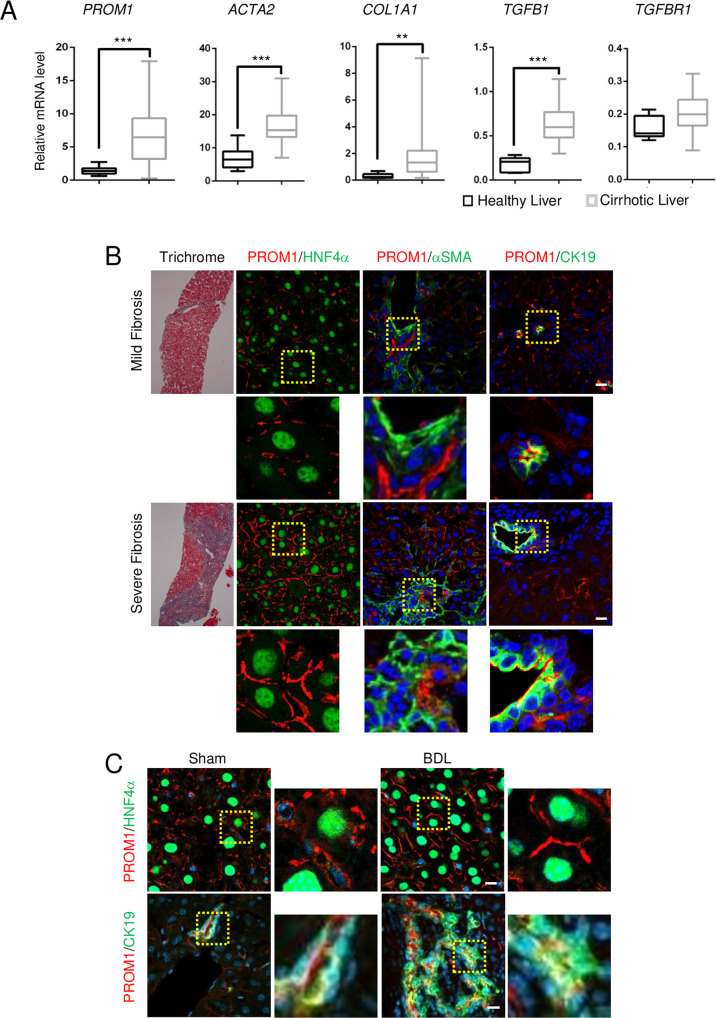

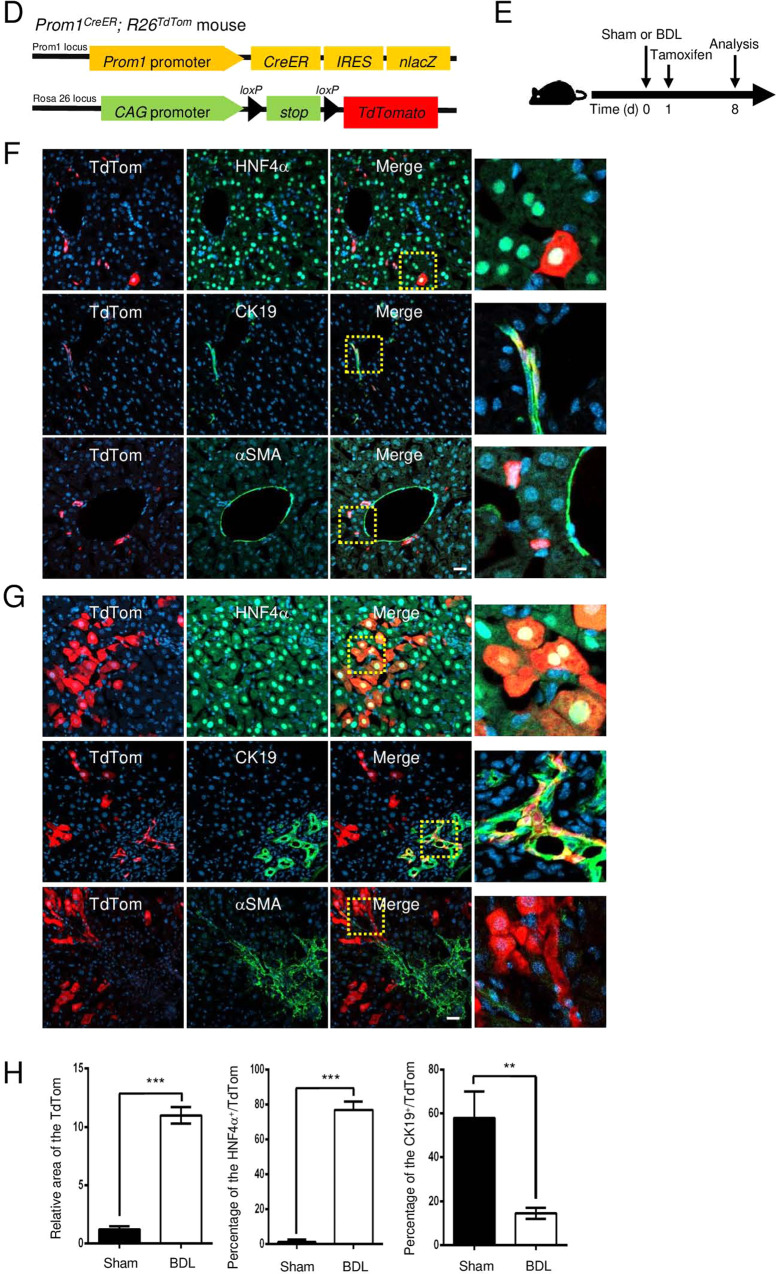
PROM1 is increased in hepatocytes and cholangiocytes of the human and mouse cirrhotic liver. **A** The relative mRNA levels of *PROM1* and fibrogenic genes (*COL1A1, ACTA2, TGFB1,* and *TGFR1*) in 6 healthy livers and 40 cirrhotic livers were obtained from a human GEO dataset (GEO accession code GSE25097) and are presented using a box-whisker plot. **B** Human liver specimens with mild and severe liver fibrosis were analyzed by Masson’s trichrome staining and double immunofluorescence analysis of PROM1 with HNF4α, αSMA, or CK19. The specimens were also stained with DAPI (blue). **C** Eight-week-old male mice were subjected to sham operation and bile duct ligation (BDL) for 7 days. Liver specimens were analyzed by double immunofluorescence analysis of PROM1 with HNF4α or CK19. The dashed boxes in the lower and right panels are enlarged. **D** *Prom1*^*CreER*^*; R26*^*TdTom*^ mice were generated and used to detect Prom1-positive cells. **E** Seven-week-old male *Prom1*^*CreER*^*; R26*^*TdTom*^ mice were injected with tamoxifen 1 day after sham surgery (*n* = 3) or BDL (*n* = 7). **F**, **G** One week after tamoxifen injection, livers were analyzed by fluorescence analysis of TdTom with immunofluorescence analysis of HNF4α, CK19, or αSMA. **H** Statistical analysis of TdTom-expressing hepatocytes and cholangiocytes in **F**, **G**. The fluorescent areas of TdTom alone (left panel), TdTom with HNF4α (middle panel), and TdTom with CK19 (right panel) were normalized to DAPI-stained dots. The dashed box in the right panel is enlarged. Scale bar = 20 µm. *t* test; **p < 0.01, ***p < 0.001. All data are the mean ± S.E.M. *ACTA2*, alpha-actin-2; *COL1A1*, alpha-1 type 1 collagen; *PROM1*, prominin-1; *TGFB1*, transforming growth factor beta 1; *TGFBR1*, transforming growth factor beta receptor 1.

The *PROM1* mRNA levels in 40 cirrhotic livers and 6 healthy livers from a human GEO dataset (GEO accession code GSE25097) were analyzed. Compared to those in healthy livers, the mRNA levels of both *PROM1* and fibrogenic genes, such as those encoding α-smooth muscle actin (*ACTA2*), α1 type 1 collagen (*COL1A1*), and transforming growth factor β1 (*TGFB1*), were upregulated in fibrotic livers, while the mRNA level of *TGFBR1* was not (Fig. 1A). Next, we determined which cells express PROM1 in livers with mild and severe fibrosis by double immunofluorescence analysis of hepatocyte nuclear factor 4α (HNF4*α* for hepatocytes), cytokeratin-19 (CK19 for cholangiocytes) or α-smooth muscle actin (αSMA for HSCs). As shown in Fig. 1B, PROM1 was found in hepatocytes and cholangiocytes but not in HSCs (Fig. 1B). Moreover, livers with severe fibrosis had a stronger immunofluorescence signal for PROM1 in the plasma membrane of hepatocytes and cholangiocytes than livers with mild fibrosis.

We also determined the tissue distribution of PROM1 and that of HNF4α or CK19 by double immunofluorescence staining of the sham and BDL livers. The immunofluorescent PROM1 signal was increased in hepatocytes and cholangiocytes in BDL livers compared with sham livers (Fig. 1C).

To demonstrate *Prom1* expression in hepatocytes, we used *Prom1*^*CreER*^*; R26*^*TdTom*^ mice whose cells expressed CreER under control of the *Prom1* promoter. We crossed the *Prom1-CreER* mice with Cre-inducible TdTom reporter mice for permanent labeling and tracing of *Prom1*-positive cells based on tamoxifen administration (Fig. 1D). TdTom-positive cells were observed in the liver 7 days after tamoxifen administration (Fig. 1E). As shown in Fig. 1F, a small number of TdTom-positive cells were found. The cells expressed CK19 or HNF4α but not αSMA, indicating that *Prom1* is expressed in the hepatocytes and cholangiocytes of the nonfibrotic liver. Next, the mice were administered tamoxifen 1 day after BDL, and TdTom-positive cells were analyzed 7 days later (Fig. 1G). TdTom-positive cells were increased 10-fold in BDL livers compared to sham livers (Fig. 1H). TdTom-positive cells in the BDL livers expressed HNF4α and CK19 but not αSMA (Fig. 1G). Because 78% of TdTom-positive cells in the BDL livers expressed HNF4α (Fig. 1H), we concluded that hepatocytes are the major cell type in which *Prom1* is upregulated during BDL-induced liver fibrosis.

### Global and liver-specific Prom1 deficiency aggravated BDL-induced liver fibrosis in mice

To understand the physiological function of PROM1 in the development of liver fibrosis, we induced liver fibrosis by BDL in global *Prom1*-deficient mice. Immunofluorescence indicated PROM1 deficiency in the *Prom*^*−*^^*/−*^ livers (KO) (Supplementary Fig. 1a). Hematoxylin and eosin (H&E) staining showed that the KO livers had more nuclei around the portal vein than the *Prom1*^+/+^ (WT) livers after BDL (Supplementary Fig. 1a). Histological analysis of collagen fibers using Masson’s trichrome and Sirius Red revealed that BDL-induced collagen deposition in the liver was increased by *Prom1* deficiency (Supplementary Fig. 1a). Immunofluorescence analysis of αSMA and CK19 showed that BDL-induced myofibroblast differentiation and ductular reaction in the liver were increased by *Prom1* deficiency (Supplementary Fig. 1a). To assess BDL-induced liver fibrosis in the WT and KO mice, we further analyzed the mRNA levels of genes associated with liver fibrosis. In the BDL mice, *Prom1* deficiency further increased the mRNA levels of TGFβ target genes (*Col1a1, Acta2, Ctgf, Pai1*, and *Timp1*), ductular reaction-related genes (*Krt19*), and cytokine genes *(Il1b, Il6*, and *Tnfa*) (Supplementary Fig. 1b). Next, to assess liver damage after BDL, the serum levels of AST, ALT and bilirubin were measured. As shown in Supplementary Fig. 1c, the serum assay showed that *Prom1* deficiency aggravated BDL-induced liver fibrosis because the serum levels of AST, ALT, and bilirubin were further increased by *Prom1* deficiency.

To determine the role of PROM1 in liver fibrosis, we generated liver-specific *Prom1*-deficient mice (*Prom1*^*f/f*^*; Alb-Cre*) by crossing *Prom1*^*f/f*^ mice (*f/f*) containing two loxP sequences flanking exon 2 of *Prom1* with Alb-Cre transgenic mice (*Alb-Cre*) (Supplementary Fig. 2a, b). Liver-specific *Prom1* deficiency was confirmed using PHs, livers, and kidneys from *Prom1*^*f/f*^*; Alb-Cre* mice and their *f/f* littermates by immunoblotting (Supplementary Fig. 2c). Indeed, *Prom1* deficiency was found in PHs and the liver but not in the kidneys of *f/f; Alb-Cre* mice.

**Fig. 2:**
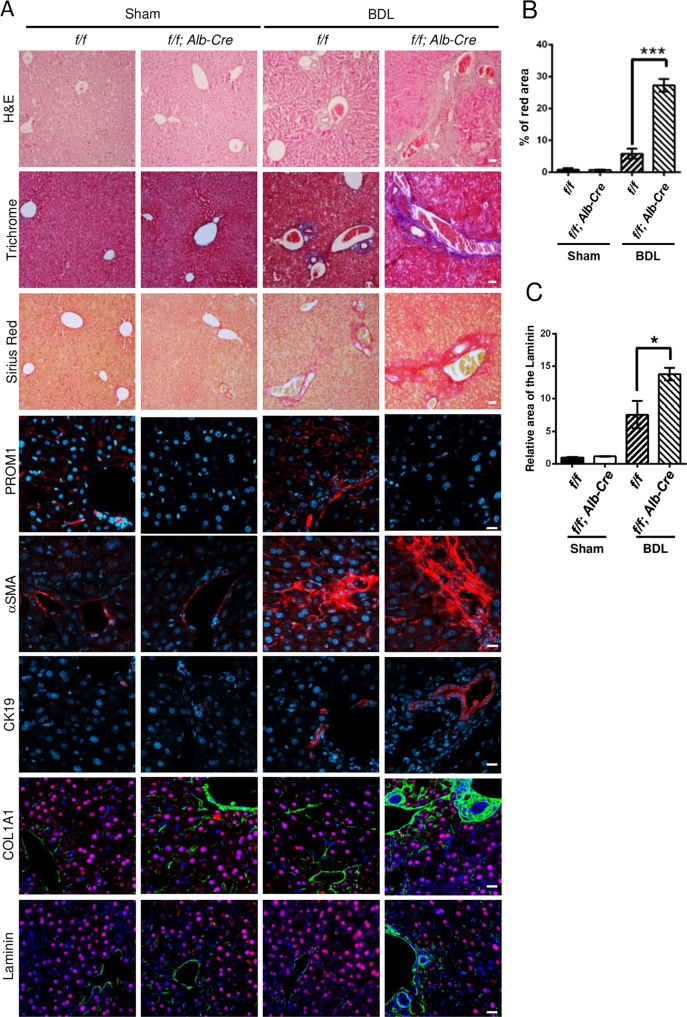

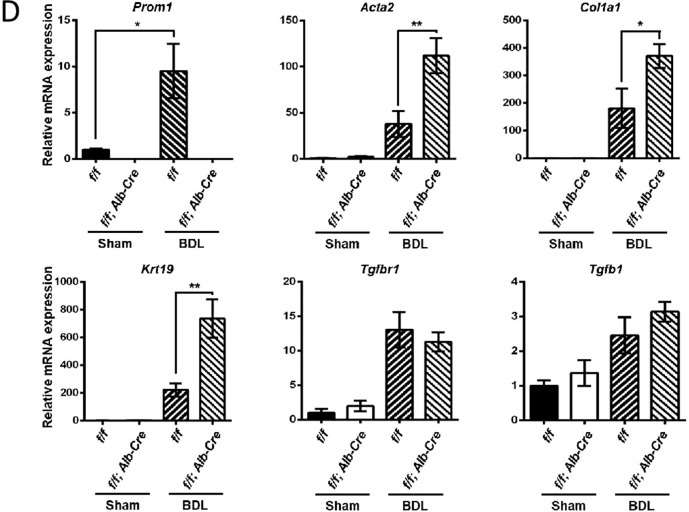
Liver-specific *Prom1* deficiency aggravated BDL-induced liver fibrosis. Eight-week-old male *Prom1*^*f/f*^ *(f/f)* and *Prom1*^*f/f*^*; Alb-Cre (f/f; Alb-Cre)* mice were subjected to sham surgery (*n* = 3) or BDL (*n* = 4–8) for one week. **A** Each liver specimen was analyzed by H&E, Masson’s trichrome and Sirius Red staining, and immunofluorescence analysis of PROM1, αSMA, and CK19. The livers were analyzed by double immunofluorescence for α1 type 1 collagen (COL1A1) or Laminin (green) with HNF4α (red). **B**, **C** Liver fibrosis was quantified by measuring the areas stained with Sirius Red (**B**) and immunostained with an anti-Laminin antibody (**C**). Three images of each liver showing Sirius Red staining or Laminin immunofluorescence were obtained. **D** The mRNA levels of *Prom1, Acta2, Col1a1, Krt19, Tgfbr1*, and *Tgfb1* were determined by RT–qPCR and normalized to those of 18S rRNA. Scale bar = 20 µm. *t* test; *p < 0.05, **p < 0.01, ***p < 0.001. All data are the mean ± S.E.M.

We next examined whether *Prom1* deficiency in the liver influences liver fibrosis in BDL mice. As shown by the results of H&E, Masson’s trichrome, and Sirius red staining (Fig. 2A), the fibrotic phenotype of the *Prom1*^*f/f*^*; Alb-Cre* mice was significantly increased compared with that of the control *f/f* mice. Indeed, liver-specific *Prom1* deficiency increased the relative area stained with Sirius Red in the BDL mice (Fig. 2B). Liver fibrosis was further analyzed by immunofluorescence analysis of αSMA and CK19. The BDL-induced expression of αSMA and CK19 was increased to a greater extent in *Prom1*^*f/f*^*; Alb-Cre* mice than in *f/f* mice (Fig. 2A). Because hepatocytes were reported to be involved in ECM deposition during liver fibrosis^24–26^, we monitored the ECM deposition area in the BDL mouse liver by immunofluorescence analysis of α1 type 1 collagen (COL1A1) and Laminin. As shown in Fig. 2A, C, *Prom1* deficiency increased COL1A1 and Laminin deposition around the ductular region but not around HNF4α-expressing hepatocytes in the BDL mouse liver. Liver-specific *Prom1* deficiency also increased the mRNA levels of TGFβ target genes (*Acta2 and Col1a1*) and a ductular reaction-related gene (*Krt19*) in BDL mice (Fig. 2D). However, similar to global *Prom1* deficiency, liver-specific *Prom1* deficiency did not change the mRNA levels of *Tgfb1* or *Tgfbr1*. These data indicated that PROM1 deficiency in hepatocytes ameliorated BDL-induced liver fibrosis by depositing ECM in the ductular region.

### Cholangiocyte-specific Prom1 deficiency did not affect BDL-induced liver fibrosis

To understand the role of cholangiocytic *Prom1* in liver fibrosis, we generated cholangiocyte-specific *Prom1*-deficient mice (*Prom1*^*f/f*^*; Krt19-Cre*) by crossing *Prom1*^*f/f*^ mice (*f/f*) with transgenic *Krt19-Cre* mice (Supplementary Fig. 3a). The PCR-based genotype of each mouse was determined from the tail tip of WT, *Prom1*^*f/f*^ (*f/f*), and *Prom1*^*f/f*^*; Krt19-Cre* (*f/f; Krt19-Cre*) mice (Supplementary Fig. 3b). Indeed, immunofluorescence analysis confirmed that the expression of PROM1 disappeared in the CK19-positive cells of *Prom1*^*f/f*^*; Krt19-Cre* livers (Supplementary Fig. 3c).

**Fig. 3:**
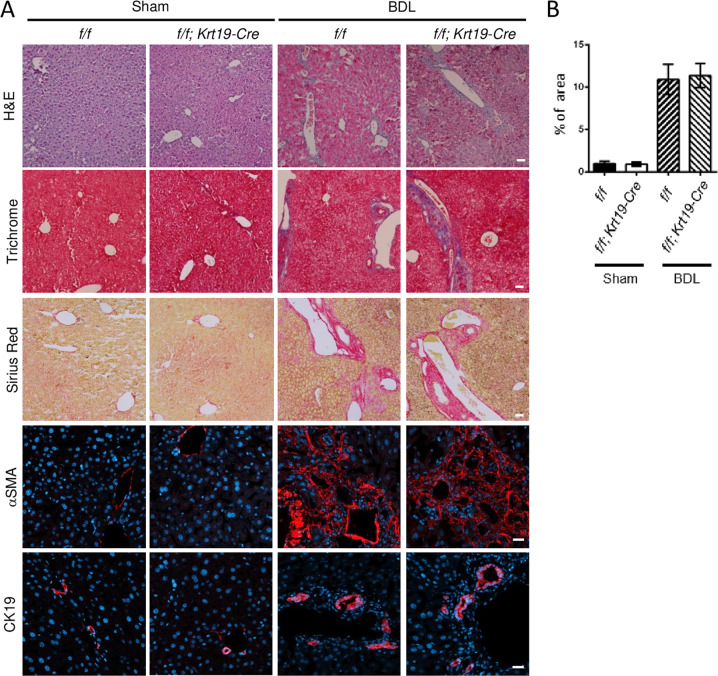

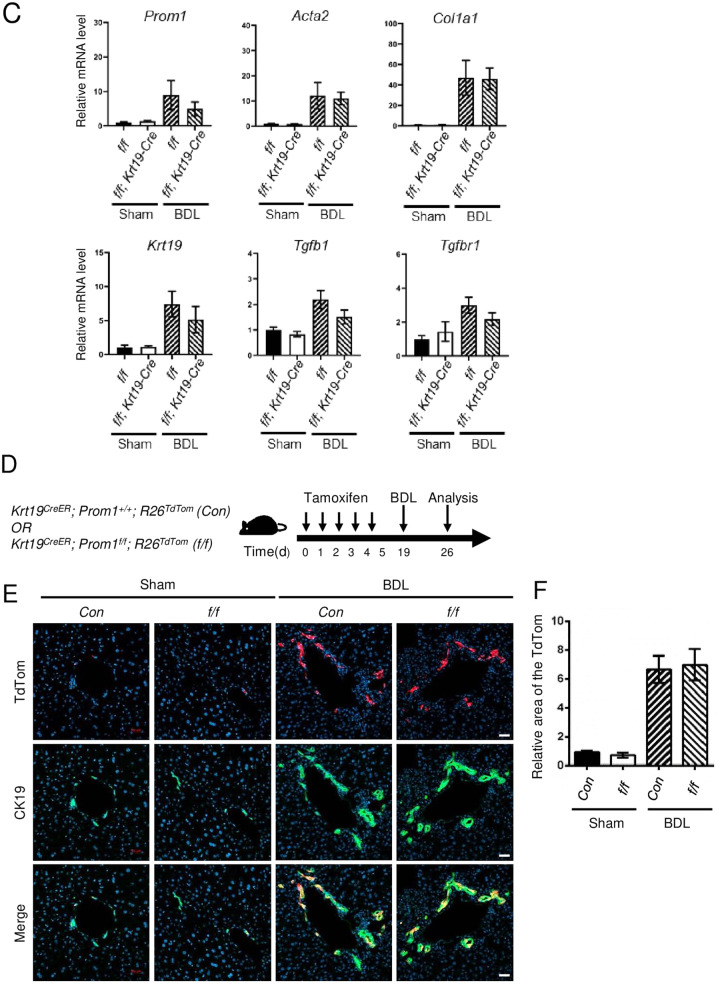
Cholangiocyte-specific Prom1 deficiency did not affect BDL-induced liver fibrosis. **A**–**C** Eight-week-old male *Prom1*^*f/f*^ *(f/f)* and *Prom1*^*f/f*^*; Krt19-Cre (f/f; Krt19-Cre)* mice were subjected to sham surgery (*n* = 3) or BDL (*n* = 7–9) for one week. **A** Each liver specimen was subjected to H&E, Masson’s trichrome and Sirius Red staining, and immunofluorescence analysis of αSMA and CK19. **B** Liver fibrosis was quantified by measuring the areas stained with Sirius Red. Two to three images of each liver showing Sirius Red staining were obtained. **C** The mRNA levels of *Prom1, Acta2, Col1a1, Krt19, Tgfbr1*, and *Tgfb1* were determined by RT–qPCR and normalized to those of 18S rRNA. **D**–**F** *Krt19*^*CreER*^*; Prom1*^*+/+*^*; R26*^*TdTom*^ (*Con*) and *Krt19*^*CreER*^*; Prom1*^*f/f*^*; R26*^*TdTom*^ (*f/f*) mice were generated for the lineage tracing of CK19-expressing cells. Six-week-old male mice were injected with tamoxifen. Two weeks after injection, the mice were subjected to sham surgery (*n* = 3) and BDL (*n* = 7) for 1 week (**D**). Each liver specimen was analyzed by TdTom fluorescence analysis and CK19 immunofluorescence analysis (**E**). Statistical analysis of TdTom-expressing cells in **E**. The TdTom fluorescence area was normalized to DAPI-stained dots (**F**). Scale bar = 20 µm. All data are the mean ± S.E.M. Con, control.

We analyzed liver fibrosis in *Prom1*^*f/f*^ *(f/f*) and *Prom1*^*f/f*^*; Krt19-Cre* (*f/f; Krt19-Cre*) mice after BDL. H&E, Masson’s trichrome, and Sirius Red staining showed that the number of nuclei around the portal vein and collagen deposition after BDL were not changed by cholangiocyte-specific *Prom1* deficiency (Fig. 3A). The relative area of the liver stained with Sirius Red was not different in the *f/f* and *f/f; Krt19-Cre* mice subjected to BDL (Fig. 3B). αSMA and CK19 immunofluorescence showed that the ductular reaction in the liver after BDL was similar in *f/f* and *f/f; Krt19-Cre* mice (Fig. 3A). The mRNA levels of *Acta2, Col1a1, Krt19, Tgfb1,* and *Tgfbr1* in the liver after BDL did not differ between *f/f* and *f/f; Krt19-Cre* mice (Fig. 3C). Interestingly, *Prom1* mRNA levels in the liver after BDL were increased in cholangiocyte-specific *Prom1*-deficient mice (Fig. 3C), suggesting that *Prom1* is upregulated in hepatocytes as well as cholangiocytes of the BDL liver.

We also analyzed the BDL-induced ductular reaction in cholangiocyte-specific TdTom-expressing and *Prom1*-deficient mice (*Krt19*^*CreER*^*; Prom1*^*f/f*^*; R26*^*TdTom*^). Because TdTom is specifically expressed in only cholangiocytes, we could monitor whether cholangiocyte proliferation or expansion is dependent on PROM1. We induced liver fibrosis by BDL in tamoxifen-treated mice; tamoxifen was administered 2 weeks before BDL (Fig. 3D). One week after BDL, TdTom expression was observed in only the biliary ducts. Indeed, as shown in Fig. 3E, F, cholangiocyte-specific *Prom1* deficiency did not change the BDL-induced expansion of cholangiocytes that expressed TdTom and CK19, as determined by CK19 immunofluorescence and statistical analysis of the number of TdTom-expressing cells. This phenotype was similar to that observed in *f/f; Krt19-Cre* mice.

### Prom1 deficiency enhanced TGFβ1 signaling in the liver

**Fig. 4:**
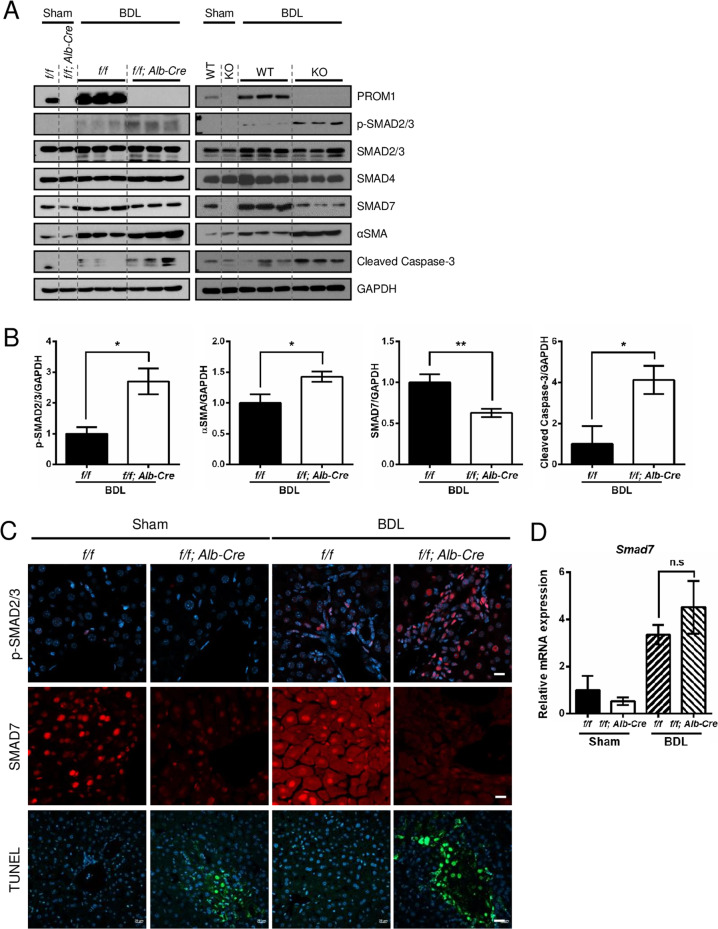
Global or liver-specific *Prom1* deficiency enhanced TGFβ1 signaling in the liver. **A**–**D** Eight-week-old male wild-type (WT), global *Prom1*-knockout (KO), *Prom1*^*f/f*^ (*f/f*), and *Prom1*^*f/f*^*; Alb-Cre (f/f; Alb-Cre)* mice were subjected to sham surgery (*n* = 3) or BDL (*n* = 7–9) for 1 week. **A** Each liver specimen was analyzed by immunoblotting for PROM1, p-SMAD2/3, total SMAD2/3, SMAD4, SMAD7, αSMA, cleaved Caspase-3, and GAPDH. *Prom1*^*f/f*^ (*f/f*) and *Prom1*^*f/f*^*; Alb-Cre (f/f; Alb-Cre)* mice are shown in the left panel. Wild-type (WT) and global *Prom1* knockout (KO) cells are shown in the right panel. **B** Band intensities of p-SMAD2/3, αSMA, SMAD7, and cleaved Caspase-3 in the livers of *f/f* and *f/f; Alb-Cre* mice subjected to BDL were statistically analyzed after normalization to the band intensity of GAPDH. **C** Liver specimens from *f/f* and *f/f; Alb-Cre* mice were analyzed by immunofluorescence analysis of p-SMAD2/3 and SMAD7 and the TUNEL assay. **D** The mRNA levels of *Smad7* from liver specimens from *f/f* and *f/f; Alb-Cre* mice were determined by RT–qPCR and normalized to those of 18S rRNA. *p < 0.05, **p < 0.01, n.s; nonsignificant. All data are the mean ± S.E.M. TUNEL, terminal deoxynucleotidyl transferase (TdT)-mediated dUTP nick-end labeling.

Because global or liver-specific *Prom1* deficiency did not change the mRNA levels of *Tgfb1* or *Tgfbr1* in BDL mice (Supplementary Fig. 1b and Fig. 2d), we speculated that PROM1 regulates TGFβ downstream signal transduction. Thus, we examined TGFβ signaling in the livers of global and liver-specific *Prom1*-deficient mice after BDL. Immunoblotting for phospho-SMAD2/3 (p-SMAD2/3) showed that global or liver-specific *Prom1* deficiency increased the BDL-induced phosphorylation of SMAD2/3 (Fig. 4A, B and Supplementary Fig. 4a). Moreover, global or liver-specific *Prom1* deficiency increased the nuclear localization of p-SMAD2/3 in the BDL liver (Fig. 4c and Supplementary Fig. 4b).

We also observed that global or liver-specific *Prom1* deficiency reduced the expression level of SMAD7 in both sham and BDL mice (Fig. 4A, B and Supplementary Fig. 4a). In addition, SMAD7 immunofluorescence showed that global or liver-specific *Prom1* deficiency decreased SMAD7 expression in sham and BDL livers (Fig. 4C and Supplementary Fig. 4b). However, global or liver-specific *Prom1* deficiency did not change *Smad7* mRNA levels in BDL livers (Fig. 4D and Supplementary Fig. 4c).

Because TGFβ induces apoptosis in hepatocytes^27^, we determined whether *Prom1* deficiency affects apoptosis in the BDL liver. Global or liver-specific *Prom1* deficiency increased BDL-induced apoptosis, as determined by immunoblotting for cleaved Caspase-3 and TUNEL assays (Fig. 4A–C and Supplementary Fig. 4a, b).

**Fig. 5:**
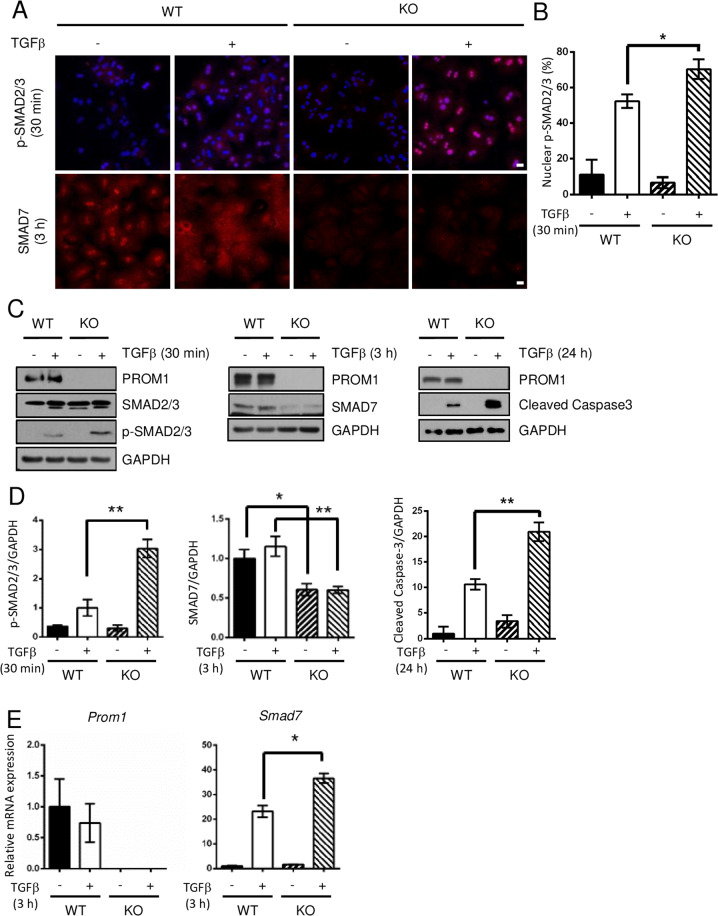

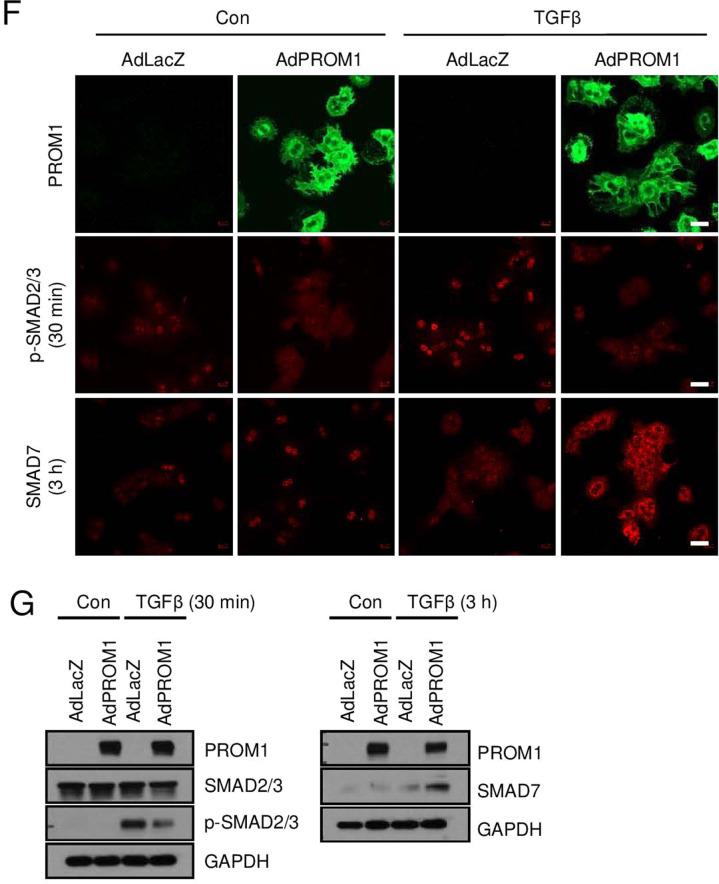
*Prom1* deficiency enhanced TGFβ signaling in hepatocytes. **A**–**E** Primary hepatocytes (PHs) were isolated from 8-week-old male *Prom1*^+/+^ (WT) and *Prom1*^−/−^ (KO) mice, grown for 4 h, serum-starved for 16 h, and treated with 5 ng/ml TGFβ for the indicated times. The cells were analyzed by immunofluorescence analysis of p-SMAD2/3 and SMAD7 (**A**); immunoblot analysis of PROM1, p-SMAD2/3, SMAD2/3, SMAD7, cleaved Caspase-3 and GAPDH (**C**); and RT–qPCR analysis of the *Smad7* and *Prom1* mRNA levels. Each mRNA level was normalized to that of 18 S rRNA (**E**). **B** The percentage of cells with nuclear p-SMAD2/3 was statistically determined (*n* = 3 for each group). **D** The band intensities of p-SMAD2/3, SMAD7, and cleaved Caspase-3 in (**C**) were statistically analyzed after normalization to the intensity of GAPDH (*n* = 3 for each group). **F**, **G** Primary KO hepatocytes were infected by adenovirus expressing LacZ or PROM1 for 24 h, serum-starved for 16 h, and treated with 5 ng/ml TGFβ for the indicated times. The expression levels and patterns of PROM1, p-SMAD2/3, total SMAD2/3 and SMAD7 were determined by immunofluorescence analysis (**F**) and/or immunoblotting (**G**). Scale bar = 20 µm. *t test*; *p < 0.05, **p < 0.01. All data are the mean ± S.E.M.

To further confirm the role of hepatocyte-specific PROM1 in TGFβ signaling, we analyzed TGFβ signaling in *Prom1*-deficient mouse PHs. *Prom1* deficiency elevated the TGFβ-induced phosphorylation of SMAD2/3 and cleaved Caspase-3 (Fig. 5A–D). Interestingly, SMAD7 protein levels were higher in WT hepatocytes than in KO hepatocytes (Fig. 5A, C, D). However, TGFβ-induced *Smad7* mRNA levels were higher in KO hepatocytes than in WT hepatocytes (Fig. 5E). We also examined TGFβ signaling after adenoviral overexpression of PROM1 in primary KO hepatocytes. Immunofluorescence and immunoblot analysis showed that the restoration of PROM1 prevented the TGFβ-induced phosphorylation of SMAD2/3 by increasing SMAD7 protein expression (Fig. 5F, G). These results indicated that PROM1 negatively regulates the TGFβ signaling pathway in PHs.

### PROM1 stabilizes the SMAD7 protein

**Fig. 6:**
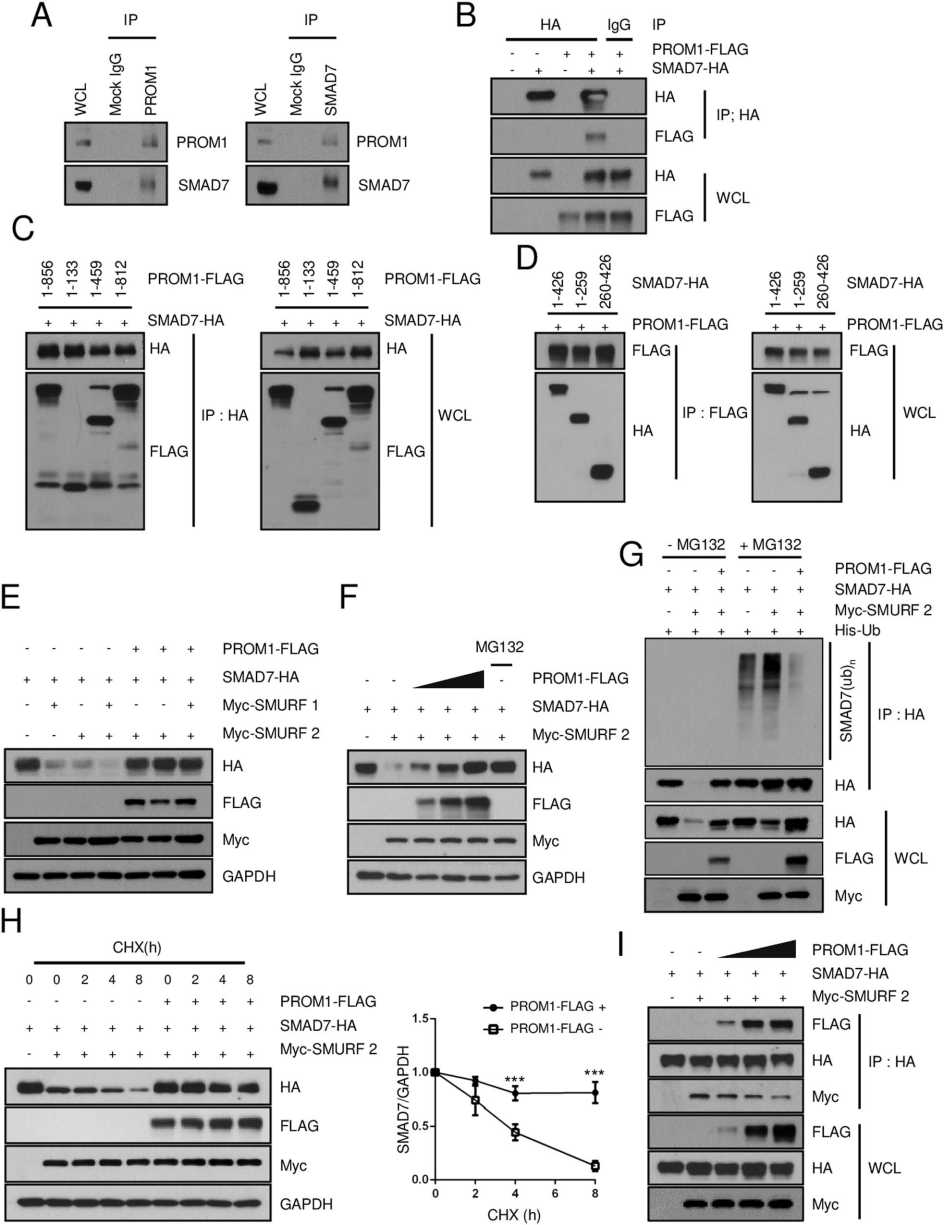
PROM1 stabilizes the SMAD7 protein. **A** The molecular association of PROM1 with SMAD7 was determined by reciprocal endogenous immunoprecipitation (IP) in the livers of 9-week-old male mice that were subjected to BDL for 1 week. **B** The molecular interaction between PROM1 and SMAD7 was determined by IP after PROM1-FLAG and SMAD7-HA were transfected into HEK293T cells. **C**, **D** Domain analysis was performed by IP after different truncated mutants of PROM1-FLAG and SMAD7-HA with the indicated combinations were used to transfect HEK293T cells. **E** After the transient expression of SMAD7-HA, Myc-SMURF-1, Myc-SMURF-2, and PROM1-FLAG at the indicated combinations in HEK293T cells, the expression level of each protein was determined by immunoblotting. **F** Different amounts of PROM1-FLAG (0, 2, and 4 μg) were transiently expressed, along with SMAD7-HA and Myc-SMURF2, in HEK293T cells. The expression level of each protein was determined by immunoblotting. MG132 (2.5 µM) treatment was used as a positive control for SMAD7-HA. **G** SMAD7 ubiquitination was determined by IP after the cotransfection of PROM1-FLAG, SMAD7-HA, Myc-SMURF2, and His-ubiquitin (His-Ub) in the presence of MG132 (2.5 µM). **H** HEK293T cells were cotransfected with PROM1-FLAG, SMAD7-HA, and Myc-SMURF2 at the indicated combinations and treated with 2 μg/ml cycloheximide (CHX) for the indicated times. SMAD7 protein levels were determined by immunoblotting (left panel). The relative protein expression of SMAD7, shown in the left panel, was statistically analyzed from three independent experiments (right panel). **I** The molecular interaction of SMAD7-HA with Myc-SMURF2 and PROM1-FLAG was assessed by co-IP after the transfection of SMAD7-HA, Myc-SMURF2, and different amounts of PROM1-FLAG (0, 2, and 4 μg). WCL, whole-cell lysate; IP, immunoprecipitation. ***p < 0.001. All data are the mean ± S.E.M.

Because the mRNA levels of *Smad7* were not significantly changed by global or liver-specific *Prom1* deficiency in BDL mice (Fig. 4D and Supplementary Fig. 4c), it is possible that SMAD7 is posttranslationally stabilized in the presence of PROM1. Thus, we examined the molecular interaction between PROM1 and SMAD7. A reciprocal endogenous immunoprecipitation assay in the BDL-treated WT liver showed a molecular interaction between PROM1 and SMAD7 (Fig. 6A). The interaction of PROM1 with SMAD7 was further demonstrated by exogenous immunoprecipitation in HEK293T cells overexpressing both PROM1 and SMAD7 (Fig. 6B). To identify the domains of each protein that are necessary for this interaction, various truncation mutants of both PROM1 and SMAD7 were prepared (Supplementary Fig. 5a, b). Coimmunoprecipitation of both the PROM1 and SMAD7 mutants showed that the 1st intracellular domain (IC1) of PROM1 interacted with the N-terminal (N) domain or Mad homology 2 (MH2) domain of SMAD7 because all PROM1 mutants with the IC1 domain interacted with all SMAD7 mutants (Fig. 6C, D). To determine whether PROM1 stabilizes SMAD7, we overexpressed different combinations of PROM1, SMAD7 and SMURF1 and SMURF2 in HEK 293T cells and monitored SMAD7 protein expression by immunoblotting. SMAD7 protein levels were decreased by SMURF2 but restored by PROM1 in a dose-dependent manner (Fig. 6E, F). In addition, SMAD7 ubiquitination induced by SMURF2 was abolished by PROM1 (Fig. 6G). The half-life of SMAD7 was determined by treating cells with cycloheximide (CHX). The half-life of SMAD7 in the presence of SMURF2 was increased 2-fold by PROM1 (Fig. 6H). To understand how PROM1 increases SMAD7 stability, we examined whether PROM1 and SMURF2 compete for interaction with SMAD7. Coimmunoprecipitation experiments showed that the molecular interaction between SMAD7 and SMURF2 was decreased by PROM1 in a dose-dependent manner (Fig. 6I). Together, these data indicated that PROM1 abolishes SMURF2-induced SMAD7 ubiquitination by interfering with the molecular interaction between SMAD7 and SMURF2.

### Adenoviral expression of PROM1 or SMAD7 ameliorated BDL-induced liver fibrosis

To confirm that the PROM1-SMAD7 axis protects against liver fibrosis, we overexpressed PROM1 by adenoviral infection in global *Prom1*-deficient mice and then analyzed BDL-induced liver fibrosis. The adenoviral overexpression of PROM1 was determined by immunoblotting of the liver (Supplementary Fig. 6a). H&E and Sirius Red staining showed that PROM1 overexpression decreased the number of nuclei around the portal vein and collagen deposition in the liver of global KO mice after BDL (Supplementary Fig. 6b, c). PROM1 overexpression also decreased the number of αSMA- or CK19-expressing cells, reduced the levels of p-SMAD2/3 and cleaved Caspase-3, and increased the levels of SMAD7 in the livers of KO mice subjected to BDL, as determined by immunofluorescence and immunoblotting (Supplementary Fig. 6b, d, e). Fibrogenic mRNA levels (*Acta2, Cola1*, and *Krt19*) determined by RT–qPCR were significantly decreased by PROM1 overexpression in the livers of KO mice subjected to BDL (Supplementary Fig. 6f).

**Fig. 7:**
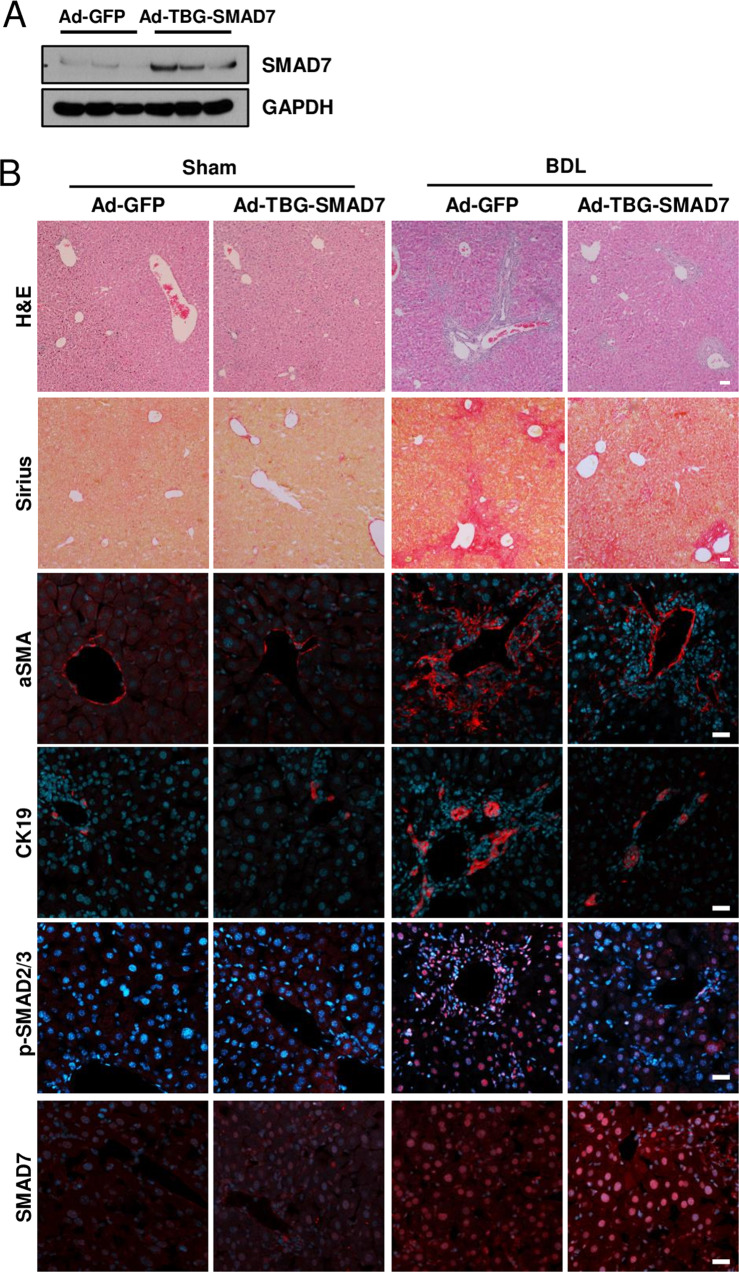

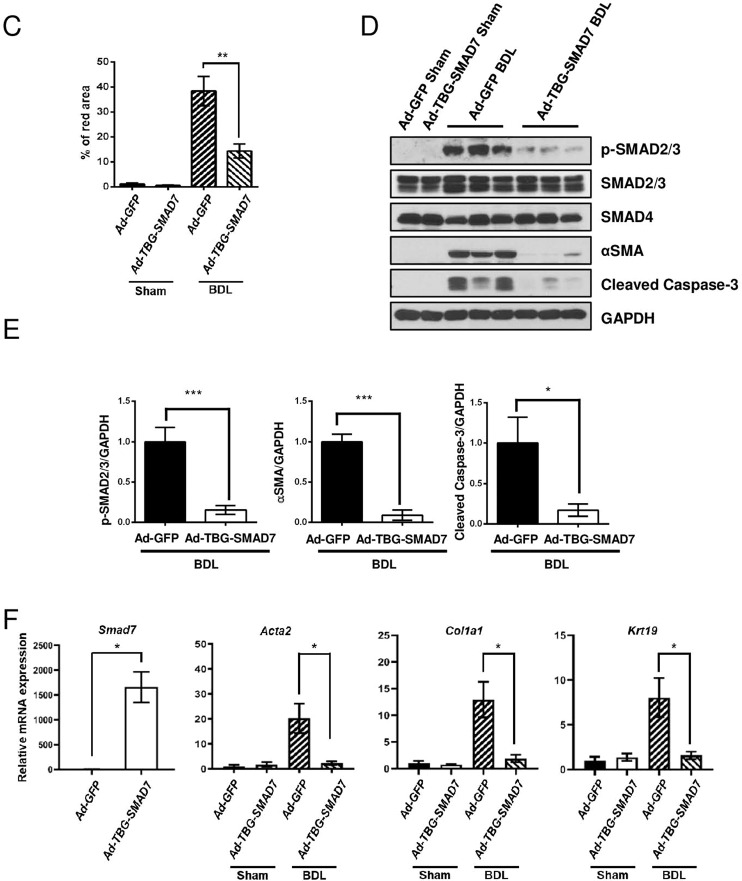
Hepatocyte-specific overexpression of SMAD7 in liver-specific *Prom1*-deficient mice ameliorated BDL-induced liver fibrosis. Eight-week-old *f/f; Alb-Cre* mice were infected by adenovirus expressing GFP or TBG-SMAD7. The mice underwent sham operation (*n* = 3) or BDL (*n* = 5–9). **A** SMAD7 expression in the liver was determined by immunoblotting 3 days after BDL. **B** Seven days after BDL, the liver was analyzed by H&E and Sirius Red staining and immunofluorescence analysis of αSMA and CK19, p-SMAD2/3, and SMAD7. **C** Liver fibrosis was quantified by measuring the area stained with Sirius Red. Two to three images of each liver showing Sirius Red staining were obtained. **D** Each specimen was analyzed by immunoblot analysis of p-SMAD2/3, SMAD2/3, SMAD4, αSMA, cleaved Caspase-3, and GAPDH. **E** The band intensities of p-SMAD2/3, αSMA, and cleaved Caspase-3 in **D** were statistically analyzed after normalization to the band intensity of GAPDH. **F** The mRNA levels of *Smad7, Acta2, Col1a1*, and *Krt19* in each liver specimen were analyzed by RT–qPCR. The mRNA levels were normalized to those of 18S rRNA. TBG, thyroxine-binding globulin. Scale bar = 20 µm. *p < 0.05, **p < 0.01, ***p < 0.001. All data are the mean ± S.E.M.

Next, we infected liver-specific *Prom1*-deficient mice with adenovirus expressing TBG-SMAD7. SMAD7 was overexpressed in only hepatocytes because the promoter of thyroxine-binding proteins (TBG) is activated in only hepatocytes^28^. Adenoviral overexpression of SMAD7 was analyzed by immunoblotting of the liver (Fig. 7A). H&E and Sirius Red staining revealed that SMAD7 overexpression alleviated BDL-induced liver fibrosis in *Prom1*^*f/f*^*; Alb-Cre* mice (Fig. 7B, C). The BDL-induced increase in the expression levels of αSMA, CK19, p-SMAD2/3, and cleaved Caspase-3 was reduced by hepatocyte-specific SMAD7 overexpression, as determined by immunofluorescence and immunoblotting (Fig. 7B, D, E). In addition, SMAD7 overexpression prevented BDL-induced upregulation of fibrogenic mRNAs (*Acta2, Col1a*, and *Krt19*) (Fig. 7F). Based on all these data, we concluded that hepatocyte-specific PROM1 plays a central role in the regulation of TGFβ signaling and liver fibrosis via SMAD7 stabilization.

## Discussion

PROM1 was highly expressed in the fibrotic livers of human patients and mice (Fig. 1). To determine whether the upregulation of PROM1 is an important regulator of liver fibrosis or simply the result of liver fibrosis, we analyzed BDL-induced liver fibrosis in global, liver-specific and cholangiocyte-specific *Prom1-*deficient mice. Because BDL-induced liver fibrosis was aggravated by global and liver-specific *Prom1* deficiency but not by cholangiocyte-specific *Prom1* deficiency, hepatocellular PROM1 was determined to be a negative regulator of liver fibrosis induced by TGFβ signaling. In our model based on the results (Supplementary Fig. 7), PROM1 negatively regulates TGFβ signaling in hepatocytes. PROM1 interacts with SMAD7 and increases SMAD7 protein expression by interfering with the molecular association of SMAD7 with SMURF2 and then preventing SMAD7 ubiquitination and degradation. In turn, SMAD7, which is stabilized by PROM1, blocks the TGFβ-induced phosphorylation of SMAD2/3. Thus, PROM1 deficiency increases apoptosis by enhancing TGFβ signaling with a reduced level of SMAD7 in hepatocytes. The increased apoptotic bodies caused by PROM1 deficiency aggravate liver fibrosis via HSC activation.

Hepatocyte-specific *Tgfbr2* deficiency (*Tgfbr2ΔHEP*) prevents choline-deficient amino acid (CDAA)-defined diet-induced steatosis, inflammation, and fibrosis^15^, and hepatocyte-specific SMAD7 expression blunts CCl_4_-induced liver fibrosis in mice^14^. However, damage-induced liver fibrosis is not changed by liver-specific *Tgfr2* deficiency^29^, challenging previous findings on the role of hepatocellular TGFβ signaling in the development of liver fibrosis. Because hepatocyte-specific SMAD7 overexpression ameliorated BDL-induced liver fibrosis in liver-specific *Prom1* deficiency (Fig. 7), hepatocellular TGFβ signaling might be required for the development of liver fibrosis. However, in addition to TGFβ signaling, we cannot exclude the possibility that PROM1 controls many other modulators of hepatocyte death in the development of liver fibrosis.

PROM1 was found to be localized in the plasma membrane of hepatocytes as well as in HPCs and cholangiocytes (Fig. 1B, C). Because the signal was not detected in the *Prom1*^−/−^ liver, the observed immunofluorescence signal was specific for PROM1 (Supplementary Fig. 1a). PROM1 was found in CK19- and HNf4α-expressing cells but not in αSMA-expressing cells in the BDL liver, as determined by double immunofluorescence. Moreover, in *Prom1*^*CreER*^*; R26*^*TdTom*^ mice in which liver fibrosis was induced in the presence of tamoxifen, TdTom-expressing cells were found to be hepatocytes and cholangiocytes but not HSCs (Fig. 1F, G). Thus, we focused on the role of PROM1 in hepatocytes, excluding its role in HSCs during liver fibrosis. In contrast, Fenlon et al. failed to observe hepatocellular *Prom1* expression after liver injury in *Prom1*^*CreER*^*; R26*^*TdTom*^ mice that were treated with tamoxifen 2 weeks before BDL^30^. Because *Prom1* upregulation in hepatocytes was induced within 48 h after liver injury (data not shown), tamoxifen should be administered immediately after liver injury to detect hepatocellular PROM1.

Zagory et al. demonstrated that PROM1 promotes RRV-induced liver fibrosis in newborn mice, suggesting that upregulated PROM1 during liver fibrosis aggravates liver fibrosis^22^. In contrast, we showed that hepatocellular PROM1 protects against BDL-induced liver fibrosis. To address this discrepancy, we analyzed liver fibrosis in two different mouse models, global and liver-specific *Prom1* deficiency. We also analyzed liver fibrosis after adenoviral overexpression of PROM1 or SMAD7. All these mice consistently showed the same phenotype, indicating that the hepatocyte-specific PROM1-SMAD7 signaling axis is necessary to protect against liver fibrosis.

Hepatocyte-specific deletion of *Smad7* increases TGFβ-induced apoptosis in the liver and PHs, aggravating alcohol-induced liver injury^31^. Reciprocally, hepatocyte-specific overexpression of SMAD7 attenuates CCl_4_-induced liver damage^14^. These findings are consistent with our results, which show that PROM1 prevents TGFβ-induced apoptosis in hepatocytes by interacting with and stabilizing SMAD7. Hepatocyte-originated apoptotic bodies activate HSCs that have differentiated into myofibroblasts^32,33^. Activated HSCs are a major cell type necessary for the progression of liver fibrosis^33^. Our results suggest that hepatocyte-specific *Prom1* is necessary to attenuate TGFβ-induced liver fibrosis by stabilizing SMAD7 and reducing TGFβ-induced apoptosis in hepatocytes.

PROM1 is an important surface marker of normal and cancer stem cells and has several binding partners, such as Actin, Radixin, PI3K, and HDAC6^23,34–36^. We demonstrated that PROM1 plays a central role in TGFβ signaling and liver fibrosis by stabilizing SMAD7 using both global and liver-specific *Prom1*-deficient mice. We believe that our study will shed light on a novel physiological function of PROM1 in liver fibrosis. In addition to the liver, fibrosis progresses in other organs, such as the heart, lung, kidney, and skin, where PROM1 is highly expressed^17,37,38^. Indeed, the antifibrogenic roles of PROM1 have also been found in lung fibrosis. PROM1-expressing progenitor cells of type II lung epithelial cells were found to protect against bleomycin-induced pulmonary fibrosis^39,40^. Thus, it will be interesting to expand our understanding of the PROM1-SMAD7 interaction to explain the antifibrogenic effect of PROM1 in lung fibrosis.

## Supplementary information

Supplementary information
