# Supplementary material for: Hepatocyte-specific Prominin-1 protects against liver injury-induced fibrosis by stabilizing SMAD7
Source: Exp Mol Med. 2022 Aug 29;54(8):1277–89. doi: 10.1038/s12276-022-00831-y (PMC9440255; doi:10.1038/s12276-022-00831-y)
Supplement: Supplementary file 1 — Supplementary information [file 12276_2022_831_MOESM1_ESM.pdf]

Hepatocyte-specific Prominin-1 protects against liver injury-induced fibrosis by stabilizing SMAD7

Hyun Lee[1,2]*, Dong-Min Yu[1,2]*, Myeong-Suk Bahn[1,2]*, Young-Jae Kwon[1,2], Min Jee Um[1,2], Seo Yeon Yoon[1,2], Ki-Tae Kim[1,2], Myoung-Woo Lee[1,2], Sung-Je Jo[1,2], Sungsoo Lee[1,2], Seung-Hoi Koo[1,2] Ki Hoon Jung[3], Jae-Seon Lee[4] and Young-Gyu Ko[1,2¶]

[1]Tunneling Nanotube Research Center and [2]Division of Life Sciences, Korea University, Seoul, 02841, Korea, [3]Department of Surgery, Dongguk University College of Medicine, Gyeongju, 38067, Korea and [4]Research Center for Controlling Intercellular Communication, College of Medicine Inha University, Incheon, 22212, Korea

*These authors contributed equally.

Keywords: Prominin-1, Liver Fibrosis, Hepatocytes, TGFβ signaling and SMAD7

[¶]To whom correspondence should be addressed

Young-Gyu Ko, Ph. D.

Division of Life Sciences, Korea University

145, Anam-ro, Seongbuk-gu, Seoul, 02841, Korea

e-mail: ygko@korea.ac.kr; TEL: 82-2-3290-3453

Supplementary Figure Legends

Supplementary Table 1. Antibodies used in immunostaining and immunoblotting analysis.

Supplementary Table 2. Primers used in qRT-PCR and genotyping PCR.

Supplementary Figure 1. Global *Prom1* deficiency aggravates BDL-induced liver fibrosis. Eight-week-old male *Prom1*$^{+/+}$ (WT) and *Prom1*$^{-/-}$ (KO) mice were subjected to sham (n=3) or BDL (n=5-6) for one week. (a) Each liver specimen was analyzed by H&E, Masson's Trichrome, Sirius Red staining, and immunofluorescence for PROM1, αSMA and CK19. (b) Each liver specimen was analyzed by RT-qPCR for mRNA of *Prom1*, *Acta2*, *Col1a1*, *Krt19*, *Tgfbr1*, *Tfgb1*, *Ctgf*, *Pai1*, *Timp1*, *Il1b*, *Il6*, *and Tnfa*. The level of each mRNA was normalized to those of 18S rRNA. (c) The serum levels of aspartate transaminase (AST), alanine aminotransferase (ALT), and bilirubin were determined. Scale bar = 20 µm. *t*-test *$p < 0.05$, **$p < 0.01$, ***$p < 0.001$. All data are means ± S.E.M. *ACTA2*, Alpha-actin-2; *COL1A1*, Alpha-1 type 1 collagen; *Ctgf*, Connective tissue growth factor; *Il1b*, Interleukin 1b; *Il6*, Interleukin 6; *Pai1*, Plasminogen activator inhibitor 1; *Prom1*, Prominin-1; *Tgf1*, Transforming growth factor beta 1; *Tgfbr1*, Transforming growth factor beta receptor 1; *Timp1*, Tissue inhibitor of metalloprotease; *Tnf1*, Tumor necrosis factor 1.

Supplementary Figure 2. Preparation of liver-specific *Prom1* deficient mice (a) A scheme for the generation of liver specific *Prom1* deficient mice. Mice were created by crossing *Prom1*$^{f/f}$ mice (*f/f*), containing two loxP sequences flanking exon 2 of *Prom1*, with *Alb-Cre*

transgenic mice (*Alb-Cre*). Arrows indicate primers used for genotyping (Supplementary Table 2 shows the sequences of the primers used for genotyping PCR.). (b) Representative PCR products from tail-tip genomic DNA of WT, *f/f* and *f/f; Alb-Cre* mice. 1st, $2^{nd,}$ and 4th lanes are for the PCR products by primers of F1 and R1; 3rd and 5th lanes for those by primers of F2, R2 and internal control (*Il-6*). (c) The expression levels of PROM1 in primary hepatocytes (PHs), liver and kidney of *f/f* and *f/f; Alb-Cre* were determined by immunoblotting.

Supplementary Figure 3. Preparation of cholangiocyte-specific *Prom1* deficient mice (a) scheme for the generation of cholangiocyte-specific *Prom1* deficient mice. Mice were created by crossing $Prom1^{f/f}$ mice (*f/f*) with *Krt19-Cre* transgenic mice (*Krt19-Cre*). Arrows indicates primers used for genotyping. (b) Representative PCR products from tail-tip genomic DNA of WT, *f/f* and $Prom1^{f/f}$*; Krt19-Cre* mice. Left panel shows the PCR product by primers of F1 and R2 whereas right panel shows those with primer of F2, R2 and R3. (c) *Prom1* deficiency in cholangiocyte was determined by PROM1 and CK19 immunofluorescence. Arrows indicate CK19-staining cholangiocyte.

Supplementary Figure 4. Global *Prom1* deficiency enhances TGFβ signaling by reducing the SMAD7 level in fibrotic liver. Eight-week-old $Prom1^{+/+}$ (WT) and $Prom1^{-/-}$ (KO) were subjected to sham (n=3) and BDL (n=7) for 7 days. (a) Band intensity of p-SMAD2/3, αSMA, SMAD7 and Cleaved Caspase-3 in BDL liver of WT and KO mice (Fig. 4a) was statistically analyzed after normalized by the band intensity of GAPDH. (b) Each liver specimen was analyzed by immunofluorescence for p-SMAD2/3 and SMAD7 and TUNEL assay. (c) The mRNA levels of *Smad7* from liver specimen of WT and KO mice were determined by RT-qPCR and normalized to those of 18S rRNA. *$p < 0.05$, **$p < 0.01$, n.s; non-significant. All

data are means ± S.E.M. Scale bar = 20 μm. TUNEL, Terminal deoxynucleotidyl transferase (TdT)-mediated dUTP nick-end labeling.

Supplementary Figure 5. Truncation mutants of PROM1 (a) and SMAD7 (b) for domain analysis. EX, extracellular domain; TM, transmembrane domain; IC, intracellular domain; N domain, N-terminal domain; MH2 domain, Mad homology 2 domain.

Supplementary Figure 6. Adenoviral expression of overexpression of PROM1 in global *Prom1* deficient mice ameliorates BDL-induced liver fibrosis. Eight-week-old global *Prom1* deficient mice were infected by adenovirus with *LacZ* or *PROM1*. The mice were operated by sham (n=3) or BDL (n=5-9). (a) PROM1 expression in the liver was determined by immunoblotting 3 days after BDL. (b) Seven days after BDL, the liver was analyzed by H&E, and Sirius Red staining and immunofluorescence for αSMA and CK19, p-SMAD2/3 and SMAD7. (c) Quantification of liver fibrosis was determined by measuring areas stained by Sirius Red. 2-3 images for Sirius Red staining were obtained from each liver. (d) Each specimen was analyzed by immunoblotting for p-SMAD2/3, SMAD2/3, SMAD4, SMAD7, αSMA, Cleaved Caspase-3 and GAPDH. (e) Band intensity of p-SMAD2/3, αSMA, SMAD7 and Cleaved Caspase-3 in d was statistically analyzed after normalized by the band intensity of GAPDH. (f) Each liver specimen was analyzed by RT-qPCR for mRNAs of *PROM1, Acta2, Col1a1* and *Krt19*. The mRNA levels were normalized by those of 18S rRNA. Scale bar = 20 μm. $*p < 0.05$, $**p < 0.01$, $***p < 0.001$. All data are means ± S.E.M.

Supplementary Figure 7. Proposed model for hepatocytic Prominin-1 protects against

liver injury-induced fibrosis by stabilizing SMAD7. Prominin-1 in the hepatocytes decrease TGFβ signaling by regulating SMAD7 protein expression. PROM1 prevented SMURF2-induced SMAD7 ubiquitination and degradation by interfering with the molecular association of SMAD7 with SMURF2.

Name	Company	Host	Experiments
PROM1	eBioscience	Rat polyclonal	IB(1:2000), IHC(1:100)
PROM1	MACS	Mouse Monoclonal	IB(1:200), IFA(1:25)
PROM1	Merck Millipore	Mouse monoclonal	IB(1:1000), IFA(1:100)
PROM1	R&D systems	Mouse monoclonal	IB(1:1000), IFA(1:100)
a-SMA	abcam	Rabbit polyclonal	IB(1:1000),IHC(1:200)
CK19	abcam	Mouse Monoclonal	IB(1:1000),IHC(1:200)
SMAD2/3	Cell Signaling Technology	Rabbit polyclonal	IB(1:1000)
pSMAD2/3	Cell Signaling Technology	Rabbit polyclonal	IB(1:500), IFA(1:100)
SMAD4	Cell Signaling Technology	Rabbit polyclonal	IB(1:1000)
SMAD7	R&D systems	Mouse monoclonal	IB(1:1000), IFA(1:100)
FLAG	Sigma-Aldrich	Rabbit monoclonal	IB(1:2000)
HA	Merck Millipore	Mouse monoclonal	IB(1:1000)
Myc	Santa Cruz Biotechnology	Mouse monoclonal	IB(1:1000)
GAPDH	Santa Cruz Biotechnology	mouse monoclonal	IB(1:2000)
COL1A1	Cell Signaling Technology	Rabbit monoclonal	IFA(1:200)
Laminin	abcam	Rabbit polyclonal	IFA(1:200)

Supplementary Table 1

	Name	Forward	Reverse
qRT-PCR	*Prom1*	CTCATGGCTGGGGTTGGATT	TGAGCAGATAGGGAGTGTCCA
	Acta2	AGCTACGAACTGCCTGACGG	CGTGGATGCCCGCTGAC
	Col1a1	AGCACGTCTGGTTTGGAGAG	GACATTAGGCGCAGGAAGGT
	Tgfb1	GTCACTGGAGTTGTACGGCA	GGGGCTGATCCCGTTGATTT
	Tgfbr1	GCATTGGCAAAGGTCGGTTT	TGCCTCTCGGAACCATGAAC
	Krt19	GTGCTGGATGAGCTGACTCTG	GATCTTGGCTAGGTCGACACC
	Ctgf	AGGGCCTCTTCTGCGATTTC	CTTTGGAAGGACTCACCGCT
	Pai1	CACAGGCACTGCAAAAGGTC	TGTGCCGAACCACAAAGAGA
	Timp1	GGCATCTGGCATCCTCTTGT	TGGTCTCGTTGATTTCTGGGG
	Il1b	GCCCATCCTCTGTGACTCAT	AGGCCACAGGTATTTTGTCG
	Il6	TGATGGATGCTACCAAACTGGA	ACTCTGGCTTTGTCTTTCTTGT
	Tnfa	TGGGACAGTGACCTGGACTGT	TTCGGAAAGCCCATTTGAGT
	Smad7	GGGGGCTTTCAGATTCCCAA	GACACAGTAGAGCCTCCCCA
Genotype PCR	Cre transgene	CCAGGCTAAGTGCCTTCTCTACA	AATGCTTCTGTCCGTTTGCCGGT
	Internal control	CTAGGCCAGAGAATTGAAAGATCT	GTAGGTGGAAATTCTAGCATCATCC
	loxP	ATGGGGAGCATTGATTTGCC	TCTTTCTGCCTGGTTGCTTT

Supplementary Table 2

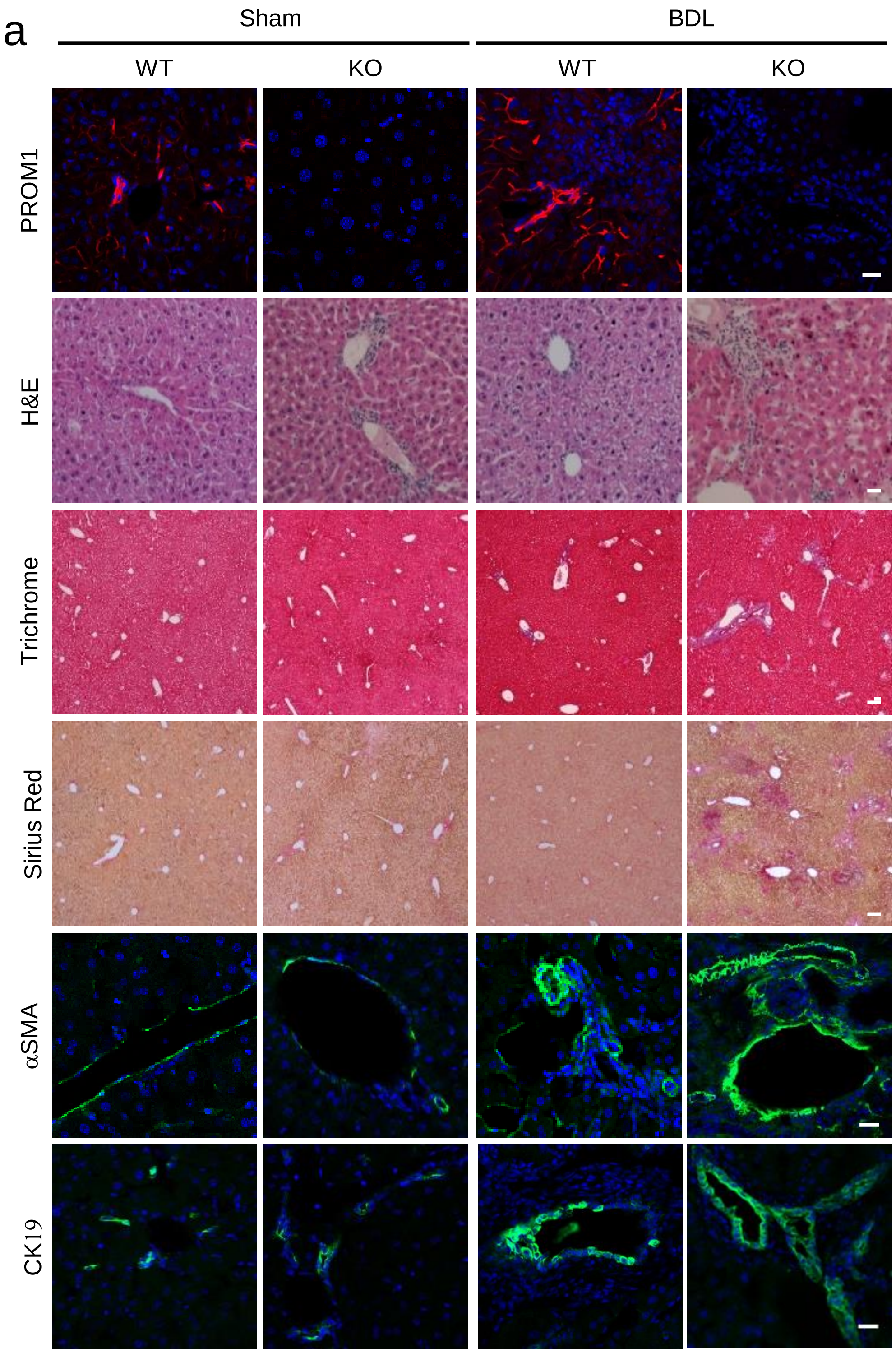

Supplementary Fig. 1

b

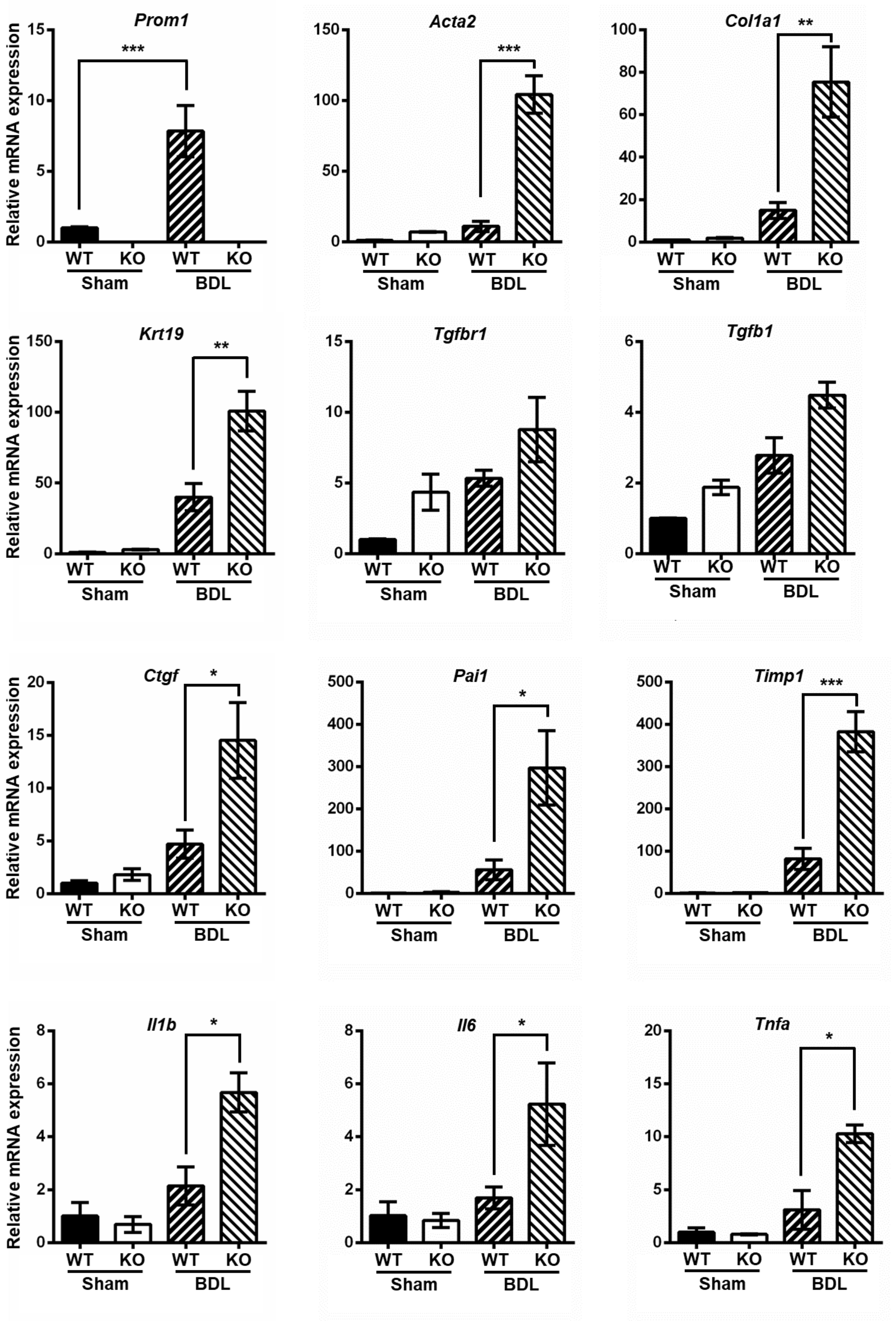

c

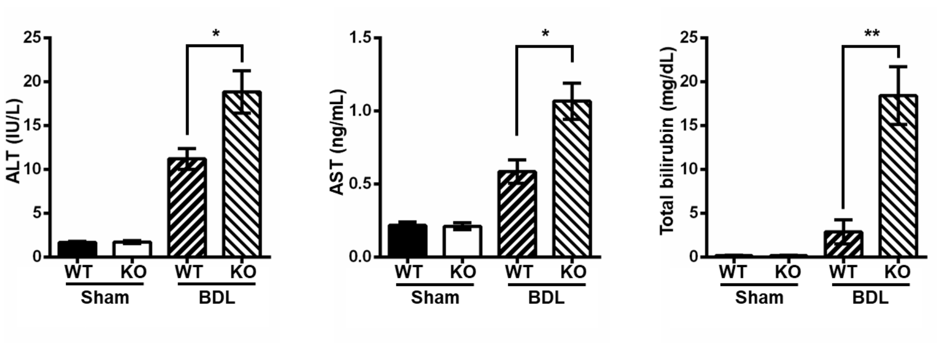

Supplementary Fig. 1, continued

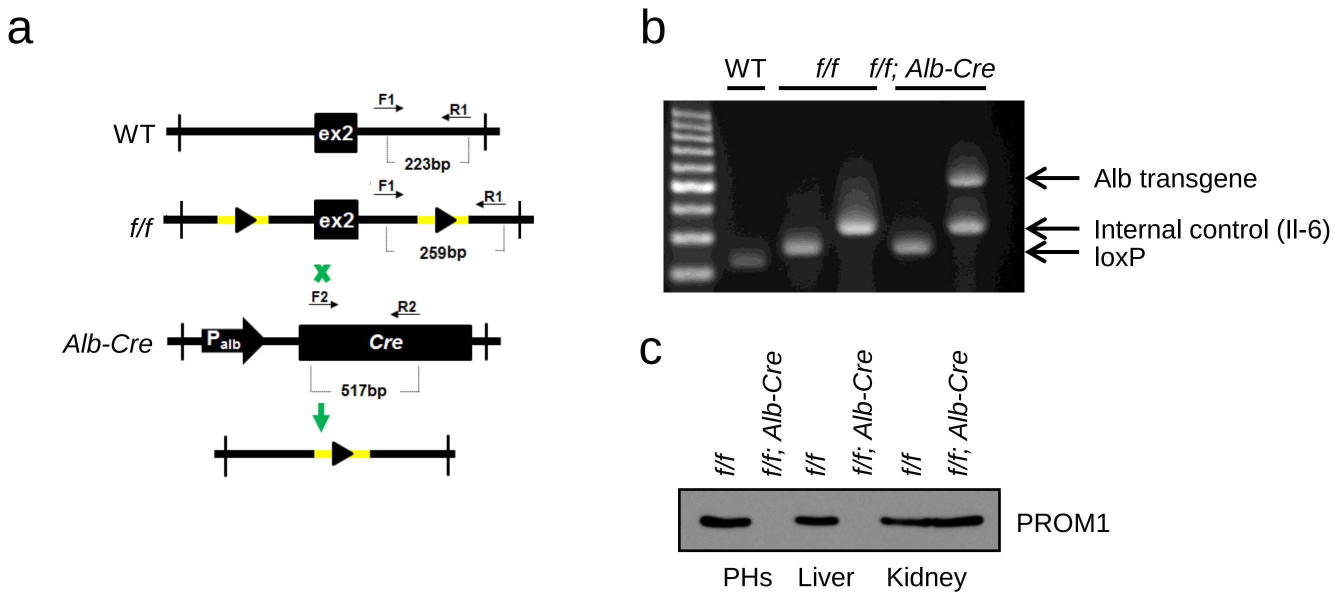

Supplementary Fig. 2

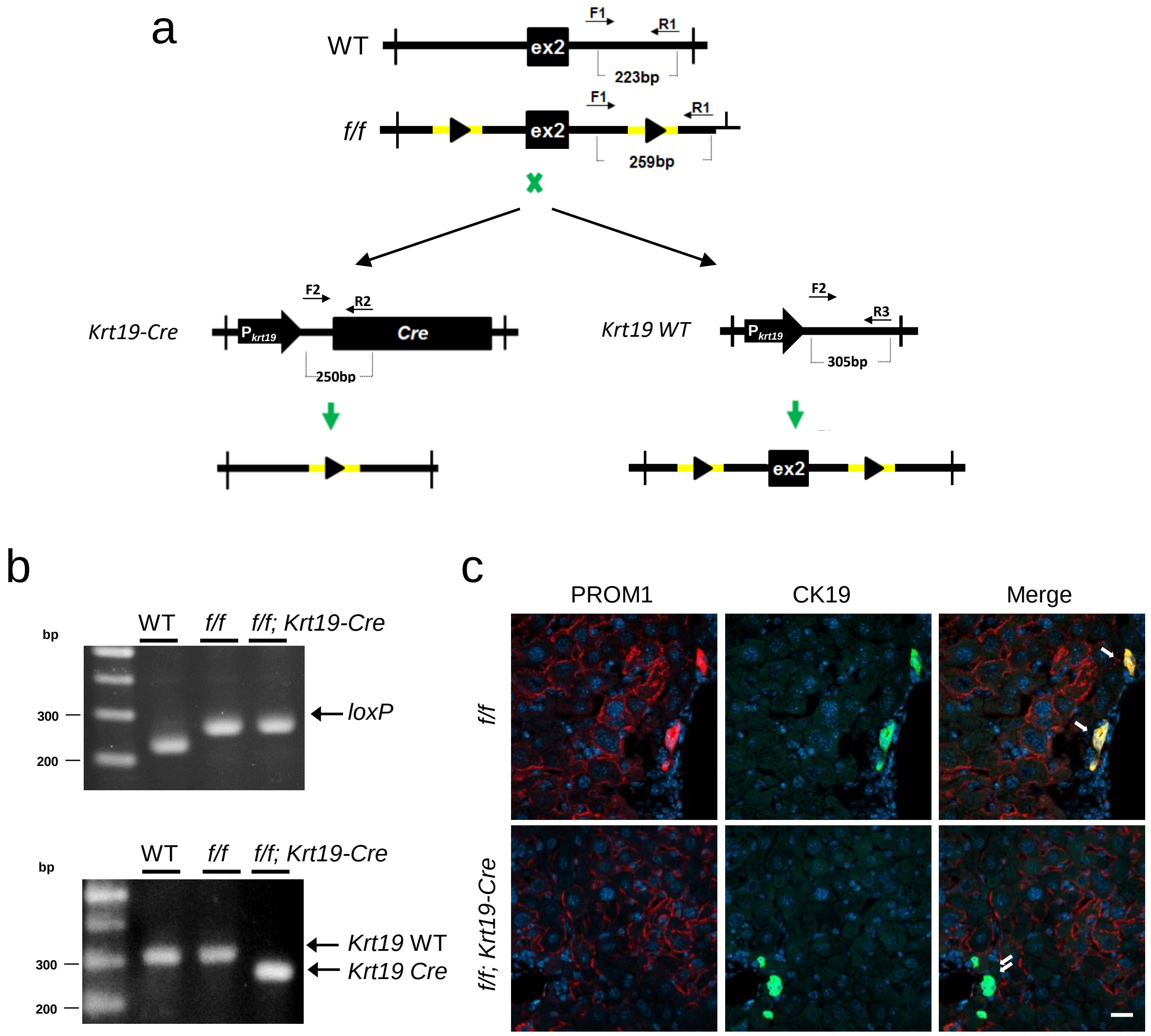

Supplementary Fig. 3

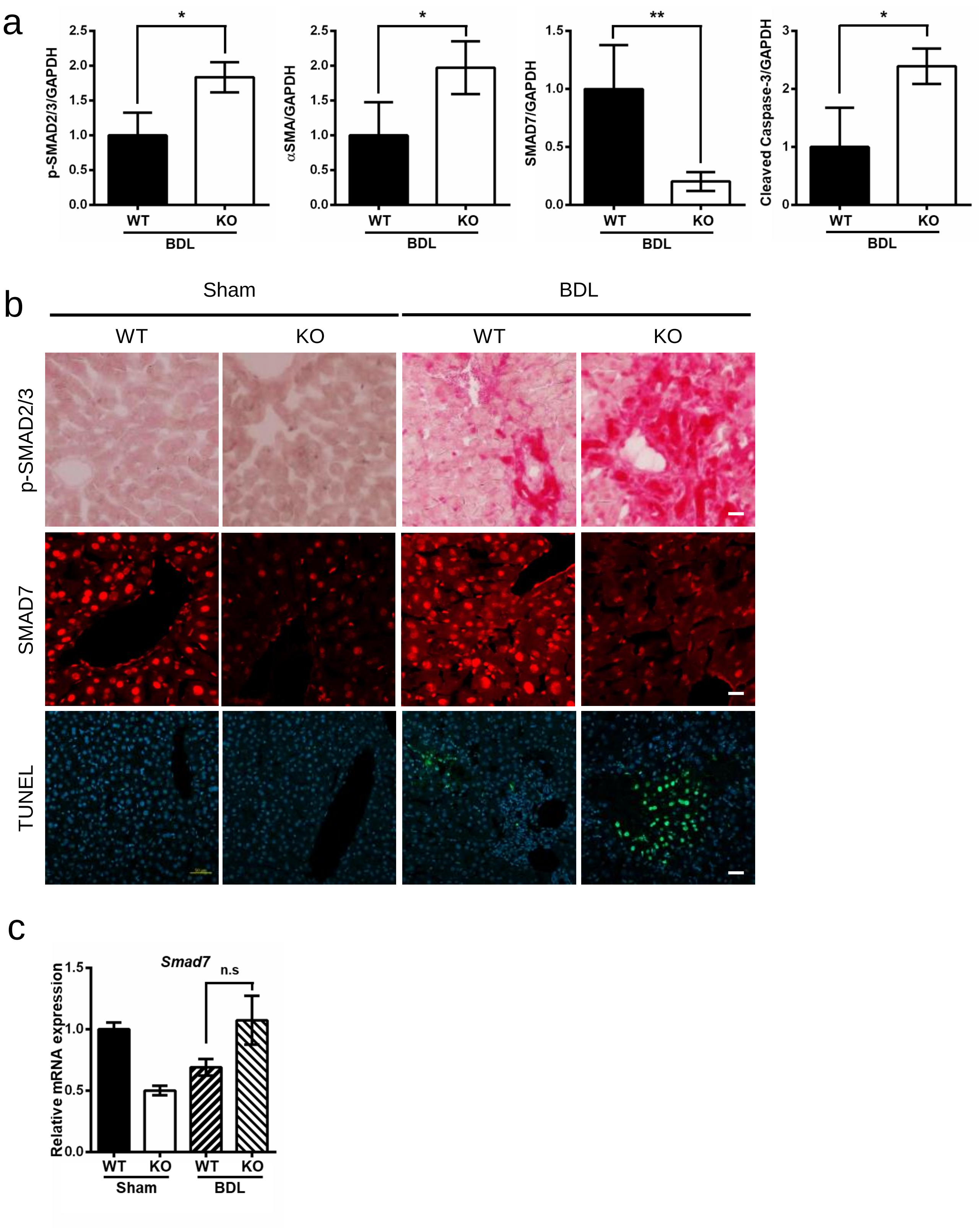

Supplementary Fig. 4

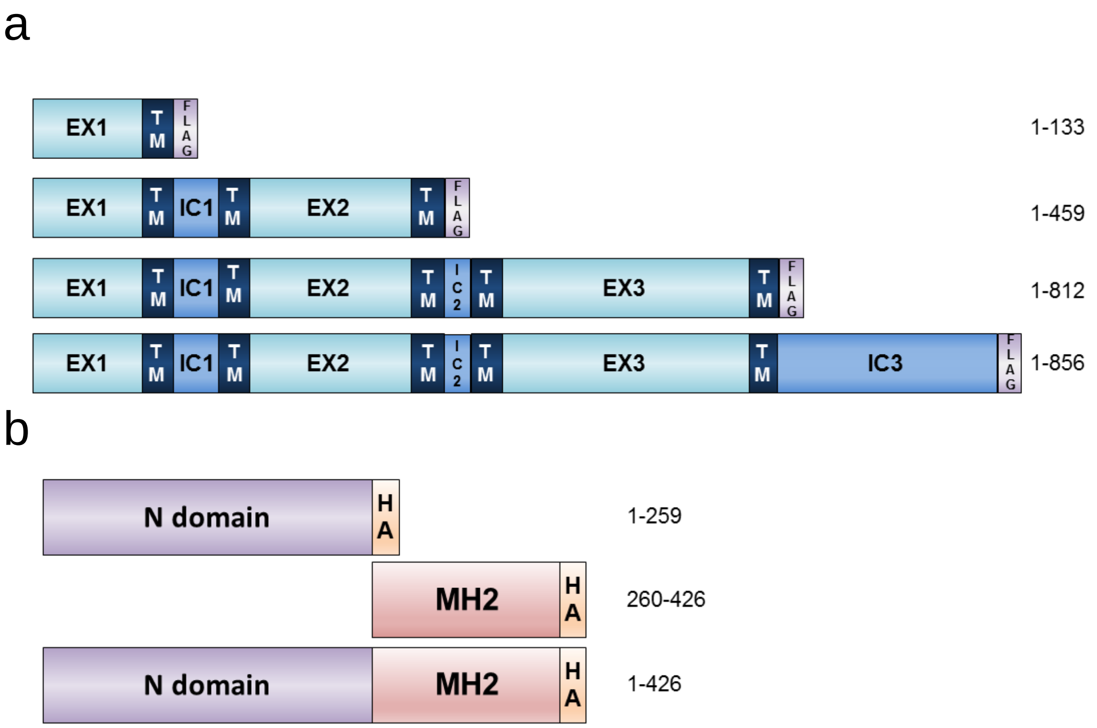

Supplementary Fig. 5

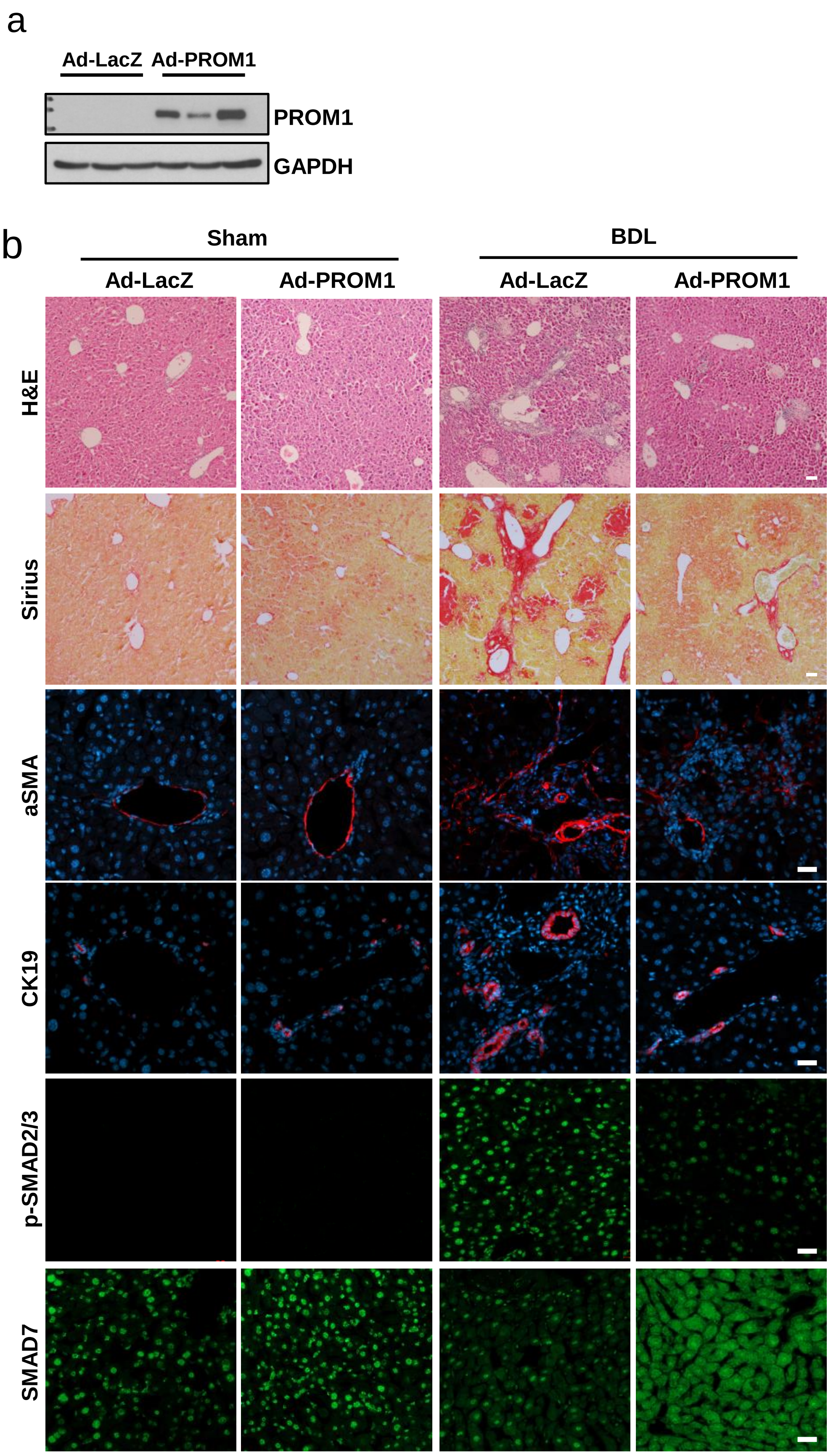

Supplementary Fig. 6

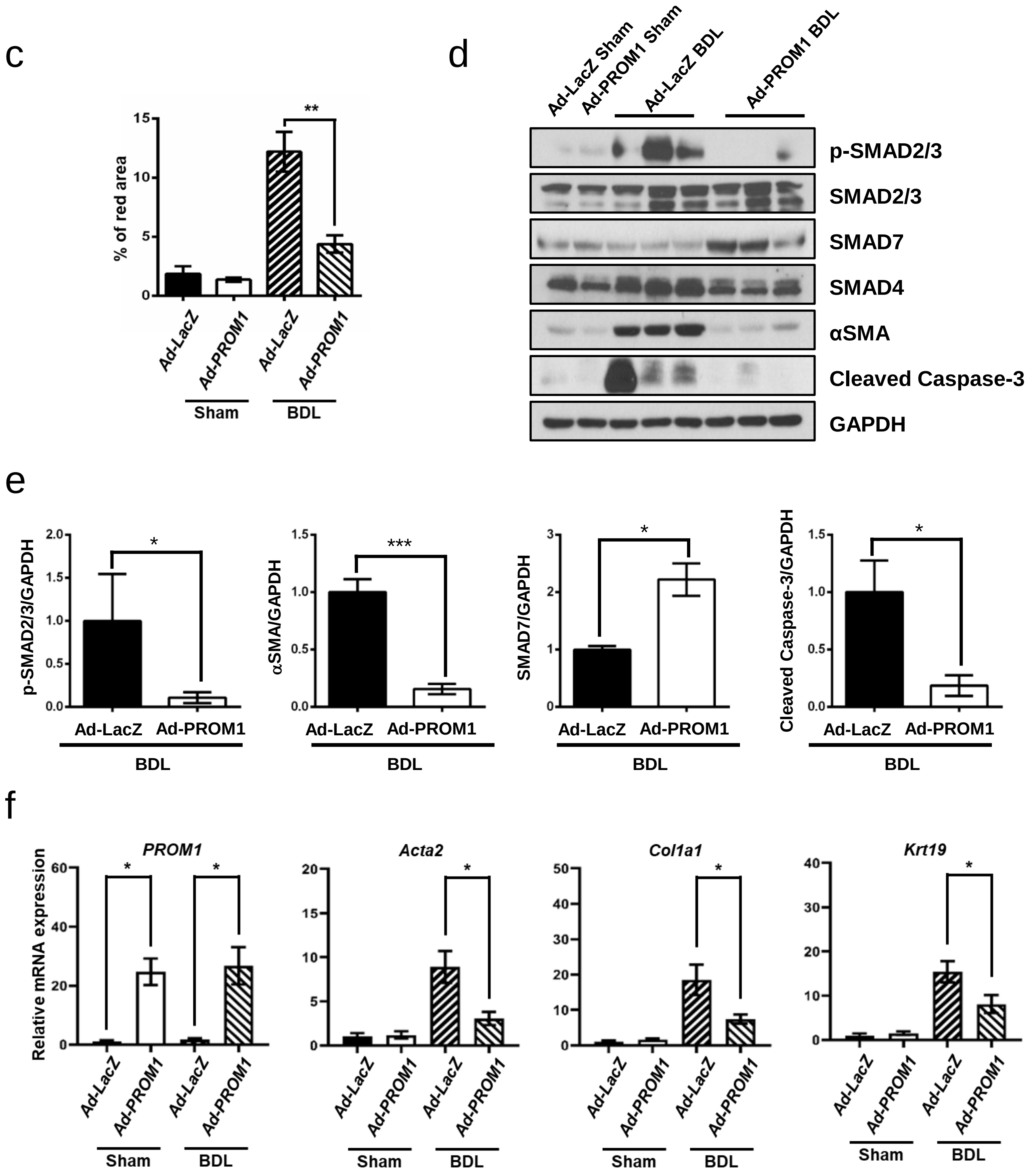

Supplementary Fig. 6, continued

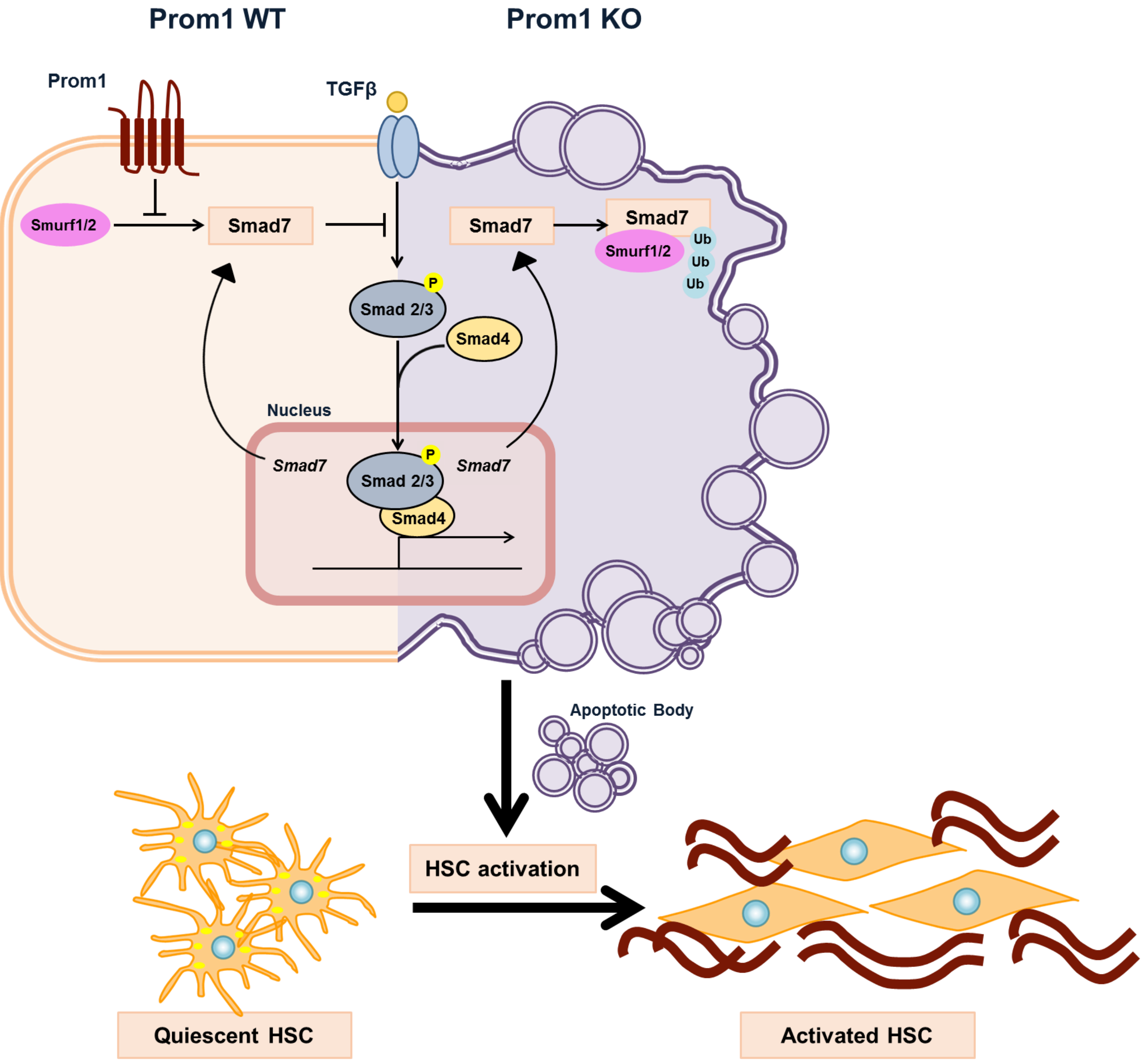

Supplementary Fig. 7
